# The impact and effectiveness of the general public wearing masks to reduce the spread of pandemics in the UK: a multidisciplinary comparison of single-use masks versus reusable face masks

**DOI:** 10.14324/111.444/ucloe.000022

**Published:** 2021-08-25

**Authors:** Ayşe Lisa Allison, Esther Ambrose-Dempster, Maria Bawn, Miguel Casas Arredondo, Charnett Chau, Kimberley Chandler, Dragana Dobrijevic, Teresa Domenech Aparasi, Helen C. Hailes, Paola Lettieri, Chao Liu, Francesca Medda, Susan Michie, Mark Miodownik, Beth Munro, Danielle Purkiss, John M. Ward

**Affiliations:** 1 UCL Plastic Waste Innovation Hub, University College London, 90 Tottenham Court Road, London W1T 4TJ, UK

**Keywords:** PPE, disposable, reusable, face mask, LCA, MFA, respirator, surgical mask, waste management, multidisciplinary comparison

## Abstract

During the coronavirus (COVID-19) pandemic, the UK government mandated the use of face masks in various public settings and recommended the use of reusable masks to combat shortages of medically graded single-use masks in healthcare. To assist decision-making on the choice of masks for future pandemics, where shortages may not be a contributing factor, the University College London (UCL) Plastic Waste Innovation Hub has carried out a multidisciplinary comparison between single-use and reusable masks based on their anatomy, standalone effectiveness, behavioural considerations, environmental impact and costs. Although current single-use masks have a higher standalone effectiveness against bacteria and viruses, studies show that reusable masks have adequate performance in slowing infection rates of respiratory viruses. Material flow analysis (MFA), life cycle assessment (LCA) and cost comparison show that reusable masks have a lower environmental and economic impact than single-use masks. If every person in the UK uses one single-use mask each day for a year, it will create a total of 124,000 tonnes of waste, 66,000 tonnes of which would be unrecyclable contaminated plastic waste (the masks), with the rest being the recyclable packaging typically used for transportation and distribution of masks. Using reusable masks creates >85% less waste, generates 3.5 times lower impact on climate change and incurs 3.7 times lower costs. Further behavioural research is necessary to understand the extent and current practices of mask use; and how these practices affect mask effectiveness in reducing infection rates. Wearing single-use masks may be preferred over reusable masks due to perceptions of increased hygiene and convenience. Understanding behaviour towards the regular machine-washing of reusable masks for their effective reuse is key to maximise their public health benefits and minimise environmental and economic costs.

## Introduction

Like many countries, the UK is currently mandating the wearing of face masks by the general public in various settings, including on public transport and in shops. This policy was implemented due to a growing body of evidence that suggested that even basic face masks can be effective in reducing the spread of the virus, by reducing the range and volume of exhaled droplets containing severe acute respiratory syndrome coronavirus 2 (SARS-CoV-2) [1]. The public health benefit of masks is based on strong epidemiological evidence [2,3]; some countries that have successfully brought the pandemic under control include China and Italy [3,4]. Hence, the World Health Organization (WHO) currently recommends this public health intervention [5].

For the general public, the UK government is advising the wearing of reusable face coverings as an alternative to the single-use face masks used in the healthcare sector. The initial reasoning for this was to ensure a sufficient supply of medical-grade masks for healthcare and frontline workers and to avoid unnecessary plastic waste. The public health basis for this decision was that, although not commonly medically certified and mostly homemade, these reusable masks are effective in combating virus transmission when combined with non-clinical interventions such as social distancing and hand washing [1,6].

In this paper, we seek to clarify some of the environmental, behavioural and economic issues around the effectiveness of daily mask wearing by millions of citizens. Specifically, we explore whether single-use masks or reusable masks would be preferable as government policy if supply issues were not a problem. We start with the anatomy of different types of masks, their material composition and effectiveness in protecting the wearer from airborne viruses. Next, we investigate the behavioural factors that impact mask use by the public and community waste management requirements. We calculate the plastic waste and environmental impact of both single-use and reusable masks and carry out a cost comparison of them both. We then discuss how these results affect the different policy options open to governments. Finally, we emphasise the need for further research to clarify the assumptions and unknowns identified in our analysis.

## European Standards, standalone effectiveness and anatomy

In the UK, face masks intended for use in the healthcare sector are regarded as personal protective equipment (PPE) and, under the EU Regulation 2016/425 on Personal Protective Equipment (the PPE Regulation), must meet the essential health and safety requirements set out in European Standards (ENs). There are two main Standards that apply to face masks. They are EN 14683:2019 ‘Medical face masks – requirements and test methods’, and EN 149:2001 ‘Respiratory protective devices – filtering half masks to protect against particles – requirements, testing, marking’. Depending on the standard, a face mask can be certified as a medical device and/or a PPE device, respectively. Masks employed in the healthcare sector meet either (or both) of the European Standards and it is conventionally single-use masks that are designed to meet the set requirements and have gained CE marking. Hence, the healthcare sector has built itself around using and managing these single-use devices, where they are treated as clinical waste and incinerated post-use. In June 2020, the European Committee for Standardisation (CEN) published CWA 17553:2020 ‘Community face coverings – Guide minimum requirements, methods of testing and use’, providing guidance on commercially available single-use and reusable masks designed for public use.

Both single-use and reusable masks have varying effectiveness in preventing human-to-human transmission of viruses. The nominal definition of a mask’s effectiveness is by its filtration efficiency – the ability to prevent particular aerosols, bacterial droplets and/or particles from penetrating the imminent atmosphere through to the mask wearer. This is their primary role amongst fulfilling other performance criteria as set out by various European Standards and the more recently published CEN Workshop Agreement on face masks, outlined above. These performance criteria as a whole aim to protect the user and not to protect others. But, as coughing and sneezing expels droplets and aerosols at high pressure, the general consensus is that face masks also protect others by capturing some, if not all, of the droplets and aerosols produced by the wearer. Penetration of droplets through the filter material, exhalation valves and around the face depends on the fit or seal of the mask [7]. It is therefore assumed that filtration efficiency is reflective of the mask’s efficacy in capturing droplets exerted by the mask wearer.

Bacteria and particle size used to test filtration efficiency vary among the standards. EN 149:2001 and EN 143:2000 require testing using the smallest particle sizes ranging from 0.2 to 2 μm (a median size of 0.6 μm), EN 14683:2019 requires the use of *Staphylococcus aureus*, a bacteria sized approximately 1 μm and CWA 17553:2020 recommends the use of 3 μm particles (the largest size). In addition, EN 149:2001, EN 140:1999 and EN 1827:1999 require adequate facial sealing and set maximum inward leakage as performance criteria. As virus particles are smaller (<200 nm) than the bacteria and particles used to test masks and filters, the classified filtration efficiencies do not convey actual efficacy against viruses.

Surgical masks (Type II and IIR) and single-use respirators (FFP2 and FFP3) are recommended for use within the healthcare sector, particularly for healthcare professionals working in environments with high risk for airborne bacteria and viruses. According to EN 14683:2019, Type I medical face masks are only recommended for use by ‘patients and other persons to reduce the risk of spread of infections particularly in epidemic or pandemic situations’ [8]. As EN 149:2001 classifies masks as PPE intended for preventing the inhalation of fine particles such as dust and pollution, the standard does not state specific recommendations concerning airborne infections. Based on filtration efficiency, masks and filters (within appropriate facepieces) classified at the highest level, under EN 149:2001 (FFP3) and EN 143:2000 (P3), respectively, provide the most protection currently available.

Unless commercially marketed for specific use, other masks are not required to comply with specific standards, and the general public can opt to use homemade or commercial reusable cloth masks that are not certified. Being uncertified means that the filtration efficiency is not confirmed. However, there are studies conveying the efficiencies of materials commonly used for making cloth masks [9,10]. As scientific evidence has emerged on the efficacies of cloth masks [10–14] and later guidelines from CWA 17553:2020, the public has gained more trust in reusable cloth masks (community face coverings and homemade/uncertified cloth masks). In Table 1 we have grouped the masks available to the general public into eight types: surgical masks, non-reusable (NR) respirators, community face coverings, reusable (R) respirators, facepieces (with replaceable filters) and homemade/uncertified cloth masks. For each mask type, we have provided the typical anatomy, the EU Standard and filtration efficiency (if applicable) and intended use. For uncertified masks, literature filtration efficiencies are quoted.

**Table 1.** A summary of the anatomy, filtration efficiency (bacterial and/or particle) and intended use information of the masks available for the general public.

Mask – referred name	Single-use (S) or reusable (R)	Typical anatomy/material	Certification standard	Performance	Testing notes	Use information
Surgical mask 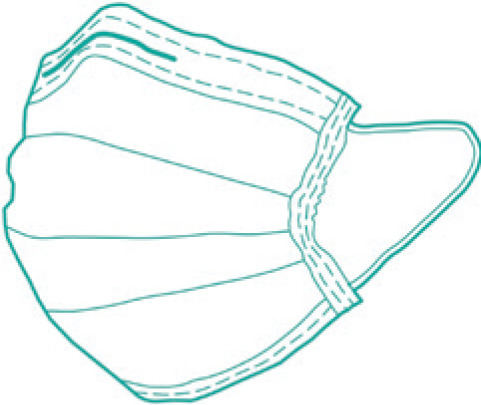	S	Multilayer of non-woven fabric typically pleated, with nose wire and ear loops Example: Layer 1 – PP non-woven spunbond Layer 2 – PP non-woven melt blown Layer 3 – PP non-woven spunbond	EN14683:2019**	BFE: Type I: >95% Type II/IIR: >98% (Type IIR is additionally splash resistant)	The bacteria size used for testing is approximately **1 μm** [8]	Both Type II and IIR are used within healthcare settings. Type I is only recommended for public use to reduce spread of pandemic.
Respirator – NR 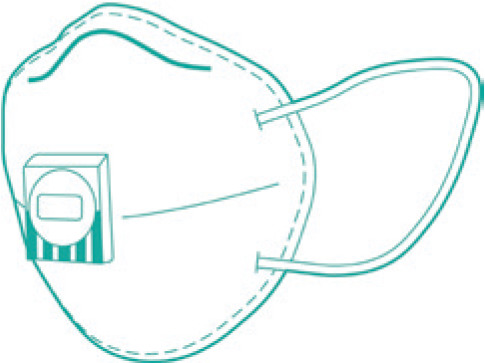	S	Multilayer of non-woven fabric typically cut to give better fit around the face, with nose wire/clip and ear loops/head straps. Can include nose foam cushioning, rubber facial seal and exhalation valve for better fit and breathability Example: Layer 1 – PP non-woven spunbond Layer 2 – cotton or PP non-woven melt blown (optional) Layer 3 – PP non-woven melt blown Layer 4 – PP non-woven melt blown Layer 5 – PP non-woven spunbond	EN149:2001**	Particle filtration efficiency/max. inward leakage: FFP1: >80%/22% FFP2: >94%/8% FFP3: >99%/2%	The particle size used for testing ranges 0.2–2 μm, with a median size of **0.6 μm** [18]	FFP3 are primarily recommended within healthcare, during the COVID-19 ‘peak’ period, UK government changed policies to allow FFP2 to be used when FFP3 are not available
Community face coverings 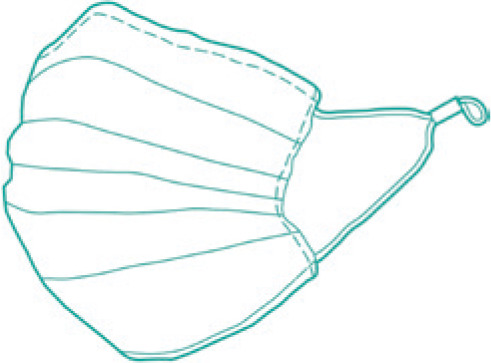	R	Bi-layer of the same material can be pleated or cut to give better fit around the face, with ear loops/head straps. Can include nose wire Materials can vary depending on intended reuse and filtration efficiency Examples: Polyester Bamboo Cotton	**EN14683:2019** **and/or** **CWA 17553:2020**	BFE: Type I: >95% Particle filtration efficiency: Level 1: >70% Level 2: >90%	The bacteria size used for testing is approximately **1 μm** [8] The particle size used for testing is approximately **3 μm** [23] Performance must be tested and maintained through to the intended number of reuses (min. 5) with 60°C washes between uses [23]	Both masks types are typically labelled with the number of reuses associated with the washing temperature Since the publication of CWA 175552:2020, the nominal washing temperature for community masks is 60°C Masks are quoted to be reusable up to 50 times (polyester)
Respirator – R 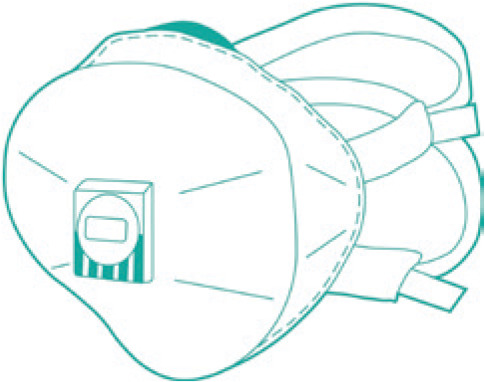	R	Multilayer like the non-reusable version but with extra structural support (layer 2), which also gives better fit. Product involves nose clip, and ear loops/head straps. Likely to include rubber facial seal and nose foam cushioning. Can include exhalation valve. Newer masks look similar to cloth masks made of polyester or cotton outer layer with inbuilt filtering materials to increase reusability Example: Layer 1 – PP non-woven spunbond Layer 2 – polymer mix (PE-PP-PET) or cotton (sturdy support layer) Layer 3 – PP non-woven melt blown Layer 4 – PP non-woven melt blown Layer 5 – PP non-woven spunbond	**EN149:2001**	Particle filtration efficiency/max. inward leakage: FFP1: >80%/22% FFP2: >94%/8% FFP3: >99%/2%	The particle size used for testing ranges 0.2–2 μm, with a median size of **0.6 μm** [5]	Although labelled as reusable, they often say reused up to two or three shifts (8 hour shift) [24]. Newer masks on the market can state up to 6 months is possible [25] Specified by the standard, manufacturer must state the cleaning protocol of the mask. Most recommend wipe cleaning (with suitable disinfectants) of the surface layers between shifts
Facepiece with designated filters 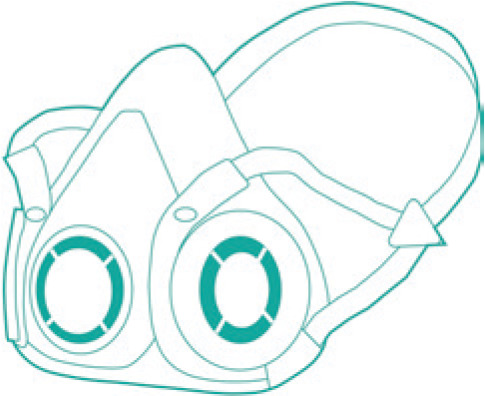	Mask: R	Mask: Typically a rigid shell/casing designed to fit as a half mask or a quarter mask made of PP, PE and/or silicone rubber. Both mask types requires exhalation valves while EN140:1999 masks are designed with an inhalation valve as well. Product involves head straps and likely requires sealant material where the facepiece meets the face. Can including cushioning for comfort	**Mask:** **EN140:1999** **EN1827:1999**	**Mask:** Max. inward leakage: 2%	Inward leakage for both masks measures leakage through the face seal [20,21]	For facepieces, manufacturers mostly recommend wipe cleaning (with suitable disinfectants) between use
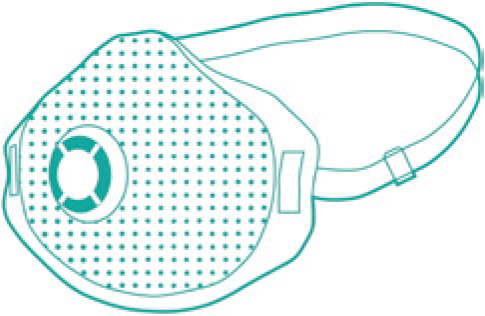	Filter: R/S	Filter: Filters are made of multilayers of non-woven spunbond and melt blown fabric. Can be either encapsulated or unencapsulated; this is dependent on the mask they are designed for Example: Layer 1 – PP non-woven spunbond Layer 2 – PP non-woven melt blown Layer 3 – PP non-woven melt blown Layer 4 – PP non-woven melt blown Layer 5 – PP non-woven spunbond	**Filter:** **EN 143:2000<thinsp>**	**Filter:** Particle filtration efficiency P1: >80% P2: >94% P3: >99%	The particle size used for testing ranges 0.2–2 μm, with a median size of **0.6 μm** [19]	For filters, they are labelled like EN149:2001 respirators where the number of shifts they can be used for is specified. Often stated to be two or three shifts
Homemade/uncertified cloth masks (may have filter pocket) 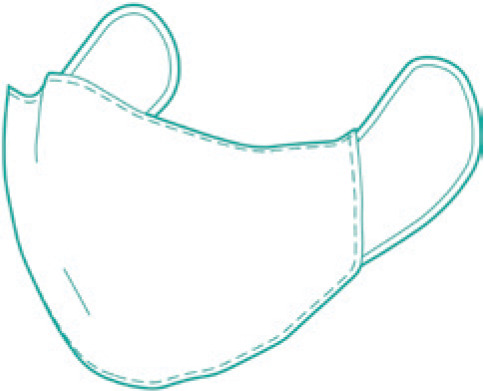	Mask: R	**Mask:** Typically two to three layers of fabric with ear loop. Can be pleated or cut to give better fit around the face. Can be made with nose wire. Can be made with a filter pocket for the insertion of filters Most common material: Cotton Most common items used: t-shirt Pillowcases	N/A	**Mask (without filter)** BFE **100% cotton t-shirt: 69.4%** **Cotton mix: 74.6%** **Pillowcase: 61.3%** Tea towel: 83.2% Vacuum cleaner bag: 94.4% Linen: 60.0% Silk: 58.0% Particle filtration efficiency: Cotton quilt: 96.1% **Cotton, 80 TPI, 2 layers: 49.0%** Cotton, 600 TPI, 2 layers: 99.5% Chiffon, two layers: 90.0% Silk, two layers: 65.0% Silk, four layers: 88.0%	Results for tests using bacteria sized **0.95–1.25 µm** are quoted. Two layers of fabric was assumed BFE for a surgical mask used in the literature study was 96.4% [9] Results for tests using particle sizes **>0.3 µm** are quoted. Particle filtration efficiency for a surgical mask (fitted correctly) used in the literature study was 99.6% [10] In bold are efficiencies of the most common materials. Note that the studies does not convey whether filtration efficiency are maintained after washes	Literature states that normal laundry processes are adequate in cleaning masks The number of reuses are not known due to limited studies. Clothing is assumed to withstand 20–50 washes [26]
	Filter: S	**Filter example: PM2.5 filter** Filters are made of multilayers of non-woven spunbond and melt blown fabric. Example: Layer 1 – PP non-woven spunbond Layer 2 – PP non-woven melt blown Layer 3 – carbon-activated filter Layer 4 – PP non-woven melt blown Layer 5 – PP non-woven spunbond	ISO 168901:2016	**PM2.5 filters** >50%	Filters claiming PM2.5 means they are sufficient in capturing particles sizes between **0.3** and **2.5 µm** [22]	Filter inserts are optional

**Note that not all surgical masks and respirators available for purchase are CE marked but may be certified according to similar standards established by other countries. Commonly referenced standards include the 42CFR84 US standard, where masks are classified to N95 (95% filter efficiency), N99 (99% filter efficiency) and N100 (99.97% efficiency) [27]. These masks are not resistant to oil unlike FFP masks. Similarly, GB2626 Chinese standard classify respirators to KN95, KN99 and KN100, which have the same filtration efficiency as the US standard [28]. N95/KN95 and N99/KN99 are deemed close equivalents to FFP2 and FFP3, respectively [29]. The modified UK government policies on face mask used within the healthcare sector allow non-CE marked single-use medical masks and respirators to be used provided that documentations of their performance meet UK and EU standards. This meant that masks certified, or in the process of getting certified, under EU and/or other countries’ standards are allowed to be used once approved by the HSE [29,30]. Filtration efficiency as required under the standard they comply to are quoted, except for homemade and uncertified commercial masks. Values from literature studies testing filtration efficiency of common fabrics following similar protocols (particularly using similar bacteria and/or particle size) as EU standards are used [8–10,18–22]. See Appendix 1 for a summary of each certification standard. BFE: bacterial filtration efficiency.

Although current standards do not require masks to be tested against viruses and virus-like particles, there are studies on evaluating the effectiveness of mask materials against them. Davies et al., Lustig et al. and Konda et al. show that common mask materials (including the surgical N95 mask) have a lower efficiency (∼5–25% reduction) in filtering viral-sized aerosols (<300 nm) compared to filtering >300 nm aerosols [9–11]. However, other studies have shown that both surgical and N95 masks are effective in preventing the transmission of common cold coronaviruses and influenza viruses from symptomatic individuals [15,16]. There is also research to suggest that simple homemade cloth masks (two-layer cotton masks) are able to limit the spread of droplets from the wearer [17]; this is evidence that cloth masks could be used to aid the prevention of transmission in public [9].

## Materials and their filtration efficiency – considerations for reusable cloth masks

**Figure 1:**
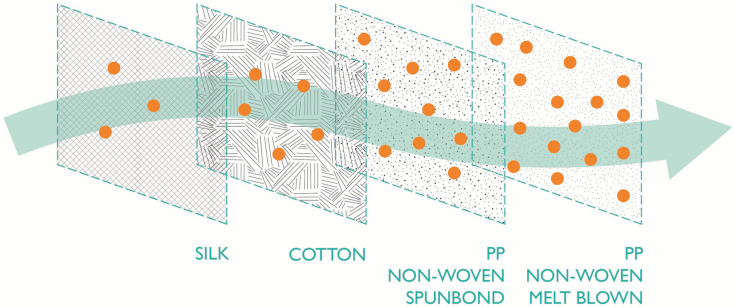
Example of filter materials in their order of filtration efficiency. Typical material filtration efficiencies increase from left to right.

The materials used to construct the filtering component of masks will determine filtration efficiency (Fig. 1). For the majority of masks, the main body of the mask acts as the filter. The exception to this is facepieces where non-porous materials form an added covering and filter. Some reusable masks also have pockets for filter inserts, which will improve their filtration efficiency. For masks without separate filters, filtration is achieved by generally layering different fabrics, or doubling (tripling) or pleating the same fabric.

Synthetic non-woven fabrics are the most common category of materials used to achieve high filtration efficiency in certified masks. These masks or filters are usually made of polypropylene (PP) but some also combine PP with polyethylene (PE) or polyester (PET) (Table 1). In the manufacturing process, heat extrusion converts the polymer into submicron diameter fibres that are collected onto a rotating belt to generate a randomly laid non-woven web [31,32]. Additional processes are used to produce webs with different structures and properties. In a spunbond process, fibres bond with each other as they cool, while in a melt-blown process, high-velocity hot air is blown on the extruded fibre to obtain ultra-fine sub-micron filaments [31–33]. The resulting melt-blown web structure typically has a smaller pore size and provides for better filtration efficiency than the spunbonded web [33]. Even so, the final filtration level will depend on the combination and specific properties of the non-woven fabric used to form the mask and/or filter component. Synthetic non-woven fabrics are often thinner and lighter in weight than other non-woven, woven and knitted fabrics. This is an additional benefit for comfort but affects durability, which is why masks made of synthetic non-woven fabrics are often single use [34,35].

Examples of the types of materials used to manufacture reusable community face coverings and homemade/uncertified cloth masks along with their filtration efficiency [9,10] are presented in Table 1. Homemade cloth masks are generally made of cotton fabric. Many DIY mask designs advise using materials such as old t-shirts [36]. Two-layer cloth masks made from 100% cotton t-shirt fabric have lower bacteria and particle filtration than the surgical masks and N95 (FFP2 equivalent) masks [9–11]. In addition to poorer filtration capacities, two-layered cotton cloth masks have a higher moisture retention than surgical masks [14]. It is recommended that single-use masks are discarded once moist, which is after up to 6 hours of use [37]. This raises the concern that cloth masks may increase the risk of infection as bacteria and viruses may be absorbed and penetrate through the mask [14]. It should be recommended that these are changed more frequently to maintain effective hygiene.

Although most reusable cloth masks have lower filtration efficiencies than N95 masks, there are some now available with filter inserts. The PM2.5 air filters (ISO 168901:2016) advertised as mask inserts comply with the ISO standard and are efficient in capturing particles with dimensions between 0.3 and 2.5 μm with >50% efficiency [22]. However, filtration efficiency is not the only important factor. A recent study suggests that inserts can decrease or improve mask performance depending on their pliability [38]. Overall, the filtration effectiveness of cloth masks depends on the fit, fineness of the cloth and the number of layers [10,11].

Another issue for reusable masks is durability. Extended use and re-use of masks means that fabric will degrade with washing, which will reduce its filtration capabilities over time. THEYA Healthcare, supplier of bamboo masks, and Isko Vital™ (Eindhoven, Netherlands), supplier of cotton masks, state that their products are washable up to 25 and 30 times, respectively [39,40]. Synthetic fabrics, such as polyesters, are widely used for community face coverings available on the market. Synthetic materials are typically water resistant, dry quicker than cotton and are more durable. Suppliers such as Decathalon (Villeneuve d’Ascq, France), Maask (London, UK) and Sera Supplies (St Albans, UK) state that their masks can be washed up to 50 times [41–43]. Maask states that their masks are CWA 17553 Level 2 compliant for up to 10 washes, and change in compliance to Level 1 for 40 washes thereafter [42]. Although masks that are made of polyester are more durable, their degradation through extended use and washing will create microfibres and microplastics. An option to reduce microplastic production would be the use of wash bags that encapsulate items in a washing machine and prevent microfibres from being discharged into the waste water [44].

Table 2 summarises the filter efficiencies of viruses and viral nanoparticles obtained by Davies et al. and Konda et al. [9,10]. The table shows that cloth masks, depending on the material combination and layers, have the potential to be more effective than the conventional surgical masks and respirators. It is acknowledged that the level of protection masks offer against viruses depends on multiple factors such as the appropriate usage and fit of the mask, level of exposure, compliance, complementary interventions (such as hand washing), and early use [14,45], amongst the type and the material of construction of the mask. Greenhalgh et al. argue that, despite the uncertainty about the efficacy of the public using cloth masks, it is better to mandate them on the grounds of the precautionary principle [46]. They acknowledge that the ability of the mask to filter viruses is only one aspect of mask efficacy, and that public behaviour around mask use is equally important. This is what we consider next.

**Table 2.** Mask material filtration efficiencies of Bacteriophage MS2 [9] and nanoparticles [10].

Material	Bacteriophage MS2 (23 nm)	Nanoparticles (<300 nm)
	Source: Davies et al. [9]	Source: Konda et al. [10]
	Filtration efficiency (%)	
Surgical mask	89.5	76
N95 (FFP2 equivalent)	–	87
Cotton		
**100% cotton t-shirt, two layers**	50.9	–
**80 TPI, two layers**	–	38
600 TPI, two layers	–	62
Cotton quilt, one layer	–	96
Cotton mix		
Unknown, two layers	70.2	–
Cotton 600 TPI × 1/silk × 2	–	94
Cotton 600 TPI × 1/chiffon × 2	–	97
Cotton 600 TPI × 1/flannel × 1	–	95
Silk		
Two layers	58	65
Four layers	–	86
Chiffon, two layers		83
Flannel, one layer		57
Other household materials, two layers		
Pillowcase	57.1–68.9	–
Scarf	48.9	–
Tea towel	72.5	–
Vacuum cleaner bag, two layers	86	–

## Behavioural considerations of single-use and reusable masks

Public behaviour towards mask use is a major contributing factor towards the overall effectiveness of masks in slowing the spread of infection [6]. Masks may be able to prevent transmission of a disease by acting as a physical barrier to aerosolised pathogens. However, correct mask use, in appropriate settings, and compliance to hygiene protocols are known to be important to prevent infection (see Appendix 2 for other recommended behaviours to prevent bacterial and viral transmission). It is hygiene protocols in particular that differ between reusable and single-use masks and the ease of adherence to the protocol by the public may make one option more effective than the other from a public health perspective.

The general hygiene guidance for mask use, which includes hand washing before applying and after removing the mask, is similar [23]. Differences lie with the post-use management of each mask type. Single-use masks are regarded as contaminated waste and, in a hospital environment, there are dedicated bins for their disposal, which typically leads to incineration. These dedicated bins do not generally exist outside a hospital environment and so it is recommended that used single-use masks are separated from general waste and double-bagged. The cleaning of reusable masks poses extra hazards in terms of contamination and hygiene. The International Scientific Forum on Home Hygiene published a report on the infection risks associated with contaminated clothing [47], which states that laundering processes eliminate the contamination from fabrics. The NHS recommends that potentially contaminated clothes should be washed at 60°C with a bleach-based product [48]. MacIntyre et al. [91] performed a randomised control trial of hospital health workers in Vietnam who both hand-washed and machine-laundered reusable masks. Their research showed that hand washing reusable masks doubled the risk of contracting seasonal respiratory viruses compared to washing the masks in the hospital laundry [49]. They also showed that properly washed cloth masks were as effective as a surgical mask. Hence, the evidence at present indicates that effective washing is important if reusable masks are to protect against viral transmission [48,49]. Given that most mask washing happens in the home, then encouraging good habits for daily machine washing of masks is important.

It would be ideal to draw on targeted behavioural analyses with regards to reusable masks and intervention evaluations amongst the target population (i.e., the general public). Unfortunately, there has been little work done on this in the literature. In the absence of high-quality evidence, we can use theories, models and frameworks from the behavioural sciences to guide analyses and extrapolate from findings across other populations and related behaviours. One such model is the COM-B model of behaviour [50,51], which posits capability, opportunity and motivation as driving influences of any given behaviour, including the effective and regular washing of masks. Capability involves both the psychological (e.g., the knowledge and importance of wearing masks) as well as the physical aspects of capability (e.g., understanding how to properly put on and wash masks). Opportunity involves both social (e.g., norms) and physical (e.g., access to masks or washing facilities), and motivation involves both ‘reflective’ (e.g., making sure the masks are washed and dried regularly) and ‘automatic’ (e.g., getting into the habit of wearing and washing masks) processes that energises behaviour. To conduct the present behavioural comparison of single-use and reusable mask use, potential behavioural influences will be summarised and compared within COM-B domains.

### Capability

Forgetfulness is a common barrier related to adherence to the behaviours associated with health (e.g., medication adherence [52,53]) and sustainability (e.g., using reusable bags when shopping [54]). Forgetting to take a mask when leaving the house is a likely barrier to mask use whether they are single-use or reusable. However, unlike the single-use plastic shopping bag scenario, single-use face masks are not readily available when one has forgotten to take one’s reusable mask. Therefore, forgetfulness is not likely to impact the use of one type of mask over and above the other. However, forgetfulness with regards to cleaning reusable masks can impact on the mask’s effectiveness under hygiene considerations. Establishing routines and habits have been identified as key behaviour change principles for reducing the spread of the viruses amongst the general public [55] and are also likely to help in combatting forgetfulness.

### Opportunity

Sociocultural paradigms can impact adherence to mask use. For instance, the contrast between mask use as a hygienic practice (i.e., in many Asian countries) versus a practice reserved for the sick (i.e., in European and North American countries) has been identified [56]. One study with German participants demonstrated that mandating, rather than encouraging mask use, caused a higher level of compliance, with other protective behaviour correlated positively [57]. It stated further that voluntary policy caused lower compliance of mask use and could intensify social stigma between people with and without masks [57]. An Italian study suggested that wearing masks causes people to comply with social distancing rules [58], adding to a growing body of evidence of this effect [59], although there are no studies yet of the differences in these behaviours that correlate with single-use or reusable masks.

The high level of compliance shown in Asian countries without mask laws may suggest that the due diligence of using a mask is dependent on cultural or social norms of individual countries. It is observed that countries that have high compliance of mask wearing by the general public also have a near-history of other epidemics that required mask wearing [1]. A study investigating influences on mask use in Japan found social norms for mask use and conformity to social norms as powerful influences on mask use behaviour [60]. Although it is unknown how transferable these findings may be to contexts outside of Asia, creating strong social norms (i.e., sense of community duty) has been identified as a key principle for enabling behaviours that will delay the spread of coronavirus (COVID-19) [61]. This can be achieved, for instance, through media and professional advice about protective action.

Availability of masks is another key consideration with respect to opportunity. Due to PPE shortages during the start of the COVID-19 health pandemic, improper use of single-use face masks was widely observed, such as not changing and reusing them, thus jeopardising their protective effect [56]. Although disinfection and reuse of single-use masks has been studied, it is not yet known how many times this can be performed before the masks become ineffective. Shortages of reusable masks are also an issue, as is the access to machine-washing facilities. No studies have yet been carried out to compare these opportunity effects on mask wearing.

### Motivation

Evidence related to other single-use and reusable hygiene products suggest that single-use may be preferable for some individuals due to the convenience it offers after use. This effect has been found with respect to menstruation products (e.g., disposable pads vs. reusable cloth pads with an antimicrobial top layer) [62]. As menstruation remains a highly taboo subject across many cultures, the generalisability of these results to the present context is questionable. However, we speculate that the considerable additional effort required from users to wash reusable masks may act as a barrier to desired adherence to mask use. Making the desired behaviour easy has been identified as a key behavioural principle for slowing the spread of COVID-19; the less effort it is to adopt a new behaviour, the more likely it is that people will do it [61].

Feeling relief from anxiety by wearing masks has also been found to promote mask use [60], although it is not clear from these results whether this effect is moderated by the type of mask worn. Perceptions of hygiene and safety may influence adherence towards use of a particular type of mask. For instance, the onset of the COVID-19 global health pandemic saw a surge in demand for food packaged in single-use packaging [63], bans on reusable cups across major café chains [64] and increased lobbying to lift bans on single-use plastic bags [65]. This suggests that an association between single-use and safety/hygiene may have entered the public psyche.

Besides hygiene and safety, it is acknowledged that environmental and cost considerations can provide motivation on which type of mask is used. The current technical guidance requires single-use masks to be discarded after one use while reusable masks are washed for reuse up to 50 times. Variability in how reusable masks are used includes the number of masks that are used in rotation; this may be influenced by their cost and subsequently influence the washing protocol followed by the user. In the scenarios below, we compare reuse models of two and four masks, and also compare machine washing with manual washing (see Table 3). Another variable is the use of additional filters within reusable masks, which is influenced by the desire to increase mask protective performance.

**Table 3.** Summary of five UK-wide face mask adoption scenarios considered in the comparative study.

Scenario number	Mask type	Mask use per day	Reuse model	Mask treatment	Number of masks per person per year	Addition of filters	Number of filters per person per year	Filter treatment
1	Single-use surgical mask	1	N/A	Disposed at the end of the day	365	No	0	N/A
2	Reusable cotton mask	1	Two masks in rotation	Manual washing, 50 washes	7	No	0	N/A
3	Reusable cotton mask	1	Two masks in rotation	Manual washing, 50 washes	7	Yes	365	Disposed at the end of day
4	Reusable cotton mask	1	Four masks in rotation	Machine washing, 30 washes	12	No	0	N/A
5	Reusable cotton mask	1	Four masks in rotation	Machine washing, 30 washes	12	Yes	365	Disposed at the end of day

We quantify the drivers of these behavioural considerations in the next sections by developing five scenarios of regular mask wearing, washing or disposal. We calculate the environmental impact and the financial costs associated with each behavioural scenario.

## Environmental assessment of face masks

Material flow analysis (MFA) and life cycle assessment (LCA) were carried out to explore the potential environmental impact of the whole UK population using either single-use masks or reusable cotton face masks for 1 year. Five UK-wide face mask adoption scenarios were modelled assuming that every person requires one mask per day (Table 3). The number of masks in use per individual per day in the UK during a pandemic depends on an individual’s responsibilities and lifestyle. Public Health England (PHE), the WHO and others, recommend surgical masks are used for maximum of 6 hours or to be discarded if found to be moist or wet (2–6 h) [66,67]. We acknowledge that the use of one mask per individual per day is likely not reflective of actual mask use. For instance, those who are exempt or shielding may not use masks and children may not wear masks as frequently as adults. However, we assumed that people who use masks more frequently (i.e., more than one per day) would balance out lower-frequency mask users. Furthermore, the average number of masks required per person per day should not affect the environmental ranking in the scenarios.

Face masks for each scenario were modelled using the cradle-to-grave approach and the functional unit (FU) employed is **1 year of mask use by the UK population (67.8 million people)** [68]. Reusable masks were modelled as made of cotton and used in rotation: if an individual has two masks, it was assumed that only manual washing was possible due to the necessary frequency of washes. With four masks, it was assumed that they could be bulk washed with normal laundry. It was assumed that masks can withstand 30 washes in the washing machine [40] and 50 washes by hand washing. All other assumptions can be found in Appendix 3.

The MFA results show that the use of reusable masks significantly reduces the amount of waste entering general waste streams (Table 4). Including the mask packaging, the total waste accumulation from using single-use masks nationally amounts to 124,000 tonnes with 66,000 tonnes of contaminated plastic. Even if single-use filters are used in addition with reusable masks, the amount of waste is >50% less than using single-use masks. There is >85% reduction in waste if reusable masks without additional filters are used.

**Table 4.** Waste arising (thousand tonnes per FU) due to face mask use in the UK for 1 year.

	S1 – single-use masks	S2 – reusable masks, manually washed, w/o filter	S3 – reusable masks, manually washed, w/ filters	S4 – reusable masks, machine washed, w/o filter	S5 – reusable masks, machine washed, w/ filters
Waste arising per FU (kt)					
Masks	66.2	6.83	6.83	11.7	11.7
Filters	–	–	29.5	–	29.5
Packaging	57.4	2.38	17.3	4.08	19.0
**Total**	**123.6**	**9.21**	**53.63**	**15.78**	**60.2**

FU; functional unit; w/o: without; w/: with.

Used (and potentially contaminated) face masks are considered medical waste and directed to incineration when they arise from a clinical setting. In the UK, there are currently 68 incinerators with a combined capacity of 12.2 million tonnes of waste [69]. In 2018, a total of 10.9 million tonnes of waste were processed [69], thus, on a national level, there is waste capacity to process the 124,000 tonnes of single-use mask waste. Currently, no specific incineration waste stream is accessible by the general public. At the household level, waste PPE is placed in mixed general waste, which may put waste collectors at risk of contracting infections. The Association of Cities and Regions for Sustainable Resource Management has advised keeping contaminated waste in a double bag for 72 hours before disposing into general waste. However, it is not clear how this would be monitored to prevent the risk to waste disposal workers. There may also be storage issues, both in households and at waste treatment sites, as the total waste increases.

**Figure 2:**
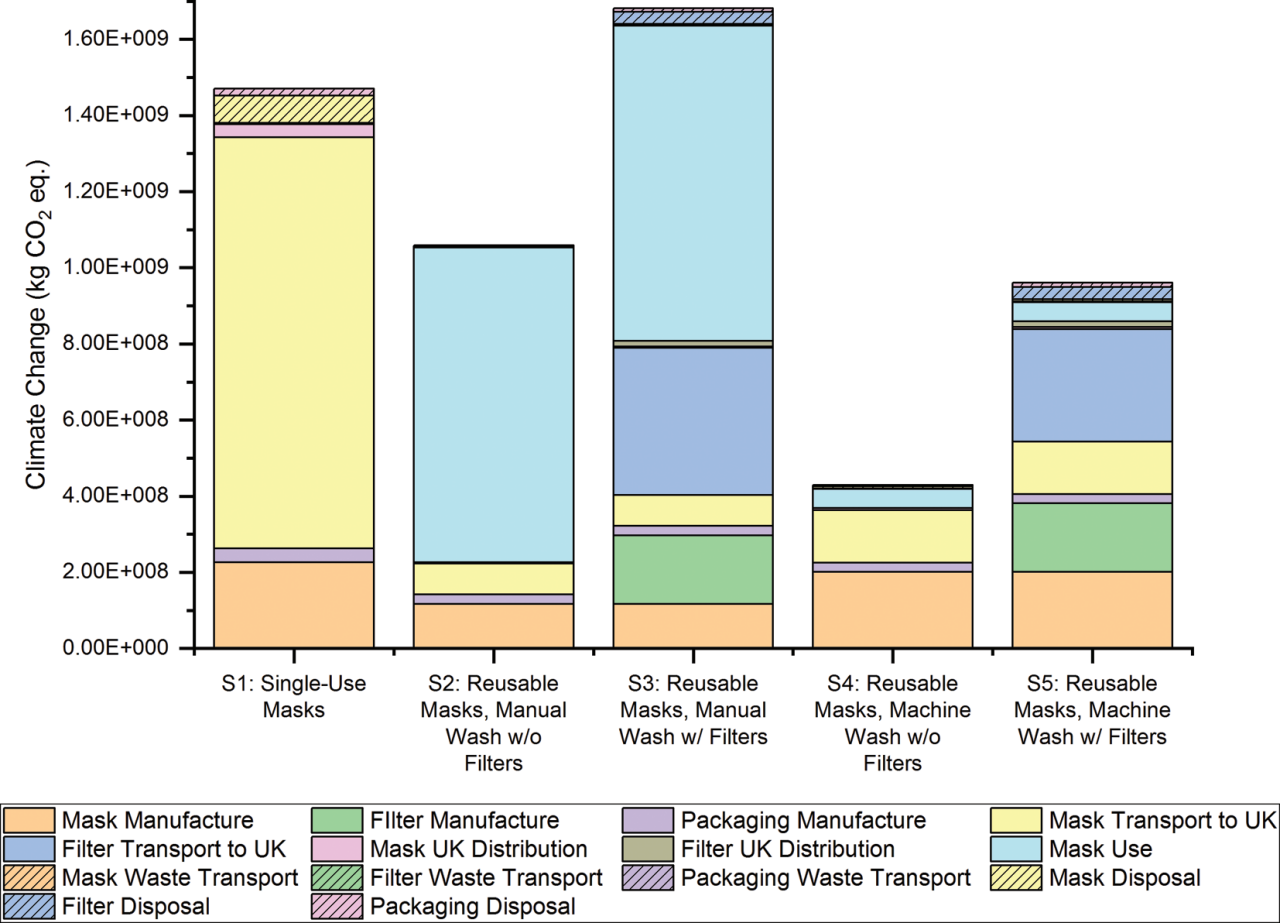
Climate change results generated for each scenario of face mask use.

Life cycle impact assessment (LCIA) results were generated using the environmental footprint (EF) 3.0 methodology [70] (see Fig. 2). It showed that Scenario 4, in which four masks are used in rotation without single-use filters and are machine-washed, has the lowest environmental impact in all of the impact categories analysed except water scarcity. Net impact results (Fig. 2 and Appendix 3) also show that having a higher number of masks in rotation to allow for machine washing (Scenarios 4 and 5) is more environmentally beneficial than manual washing (Scenarios 2 and 3).

**Figure 3:**
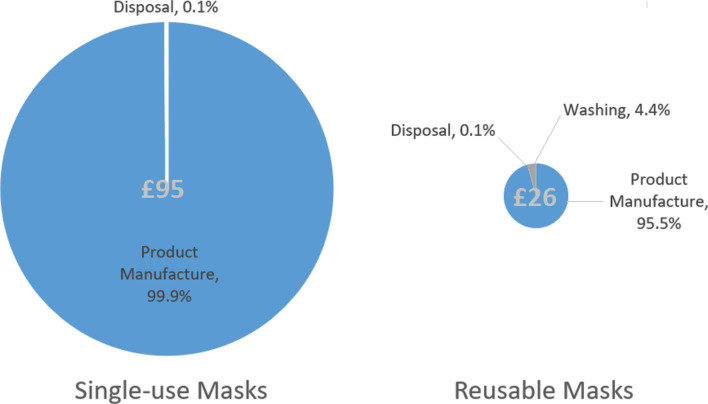
Total annual cost (£) for the supply of single-use and reusable face masks per UK citizen and the percentage breakdown.

Hot-spot analysis carried out on each scenario’s impact on Climate Change indicated that, for single-use masks, Mask Transport to the UK (from China – the assumed location for the production of all masks) contributes most to this impact category (Fig. 3). Due to the higher number of masks needed in Scenario 1, the contribution of Mask Manufacture and their Transportation to the UK is higher for this scenario than in the reusable mask scenarios. This has led to Scenario 1 generating a Climate Change result that is 3.5 times greater than Scenario 4.

In Scenarios 4 and 5, each face mask would be washed 30 times before replacement. However, the masks may not withstand this amount of washing, or they may be misplaced or damaged by other means before their assumed replacement. Further analysis indicates that if the amount of machine washes per year stays constant, up to 48 reusable masks per individual can be used before Scenario 4 exceeds the impact on Climate Change indicated by the single-use masks in Scenario 1. This analysis was carried out on all other environmental impact categories and the average cap on the supply of reusable masks was calculated to be 25 masks per person (see further details in Appendix 3).

The environmental impact of using single-use masks could be lowered by changing the manufacturing location, hence reducing transport emissions. The choice of manufacturing location would depend on the supply chain available. If the UK produced its own masks in order to reduce mask transportation emissions, as it is a major importer of non-woven textiles [71], it would still incur transport emissions from importing raw materials (explored further in Appendix 3). Importing masks from China has been deemed more realistic, owing to their manufacturing capabilities and the supply chains currently in place.

## Cost comparison

With a growing number of countries making the wearing of face masks outside of the home compulsory, price increases and limits on supply are to be expected. A cost analysis presented here compares the use of single-use and reusable masks for 1 year. Scenarios 1 and 4 assumed in the environmental impact study were used to form the basis of this cost study. Scenario 1 was chosen because it represents a single-use scenario and Scenario 4 was chosen because it generated the lowest environmental impact in most impact categories analysed (Fig. 3).

The comparison covers the initial cost of the respective product, the washing cost (including detergent and electricity) for the reusable face mask, and the disposal cost of incinerating and landfilling a single-use and a reusable face mask. The water cost was excluded from the calculations as it is typically a fixed cost in the UK (which represents no marginal variations). Transport cost is also excluded due to high variability of freight routes, modes of transport and types of vehicles required to deliver face masks to the UK population. Table 5 presents a summary of the cost comparison.

**Table 5.** Summary of cost comparison (£) between a single-use and a reusable face mask, per unit and for an annual supply.

Concept	Unitary cost (£)		Annual cost (£)	
	Single use	Reusable	Single use (365 pcs)	Reusable (12 pcs)
Product	0.26	2.04	94.99	24.43
Washing				
Detergent	N/A	0.000539	N/A	0.789
Electricity	N/A	0.000227	N/A	0.332
Disposal				
Incineration (57%)	0.000131	0.000706	0.0480	0.00847
Landfill (43%)	0.000123	0.000663	0.0450	0.00795
**Total**	**0.26**	**2.04**	**95.00**	**25.57**

### Product

The initial cost of buying any type of face mask represents the highest cost in the face mask life cycle. The unitary cost of both types of face mask were averaged from 15 products, respectively, where the products with higher relevance were selected. The average price for a single-use face mask is £0.26, and the average price for a reusable one is £2.04 [72,73], and thus a single-use face mask is around eight times cheaper. However, the annual product cost considers 365 single-use face masks (£94.99), whereas only 12 reusable masks are required (£24.43), which makes the reusable options about four times cheaper on an annual basis.

### Washing

The washing cost is only attributed to a reusable face mask. According to LCA assumptions, 0.162 g (0.156 ml) of soap are required for machine-washing one reusable mask each time. The average cost of liquid detergent (the most commonly used in the UK) is £3.46/l [74], so it will cost £0.79 to wash 12 face masks 122 times, which is the total annual cost of detergent use. Similarly, 1.58 watt-hours (Wh) are needed to wash one reusable mask once (according to LCA assumptions). The average price per kWh in the UK is £0.14 [75], and so the electricity cost of washing 12 masks 122 times results in £0.33.

### Disposal

Given that waste PPE is conventionally placed in mixed general waste at a household level, both single-use and reusable face masks are assumed to follow the same end-of-life pathway. LCA assumptions suggest that 57% of face mask waste will be incinerated (with or without energy recovery), and the remaining 43% will be landfilled. In the UK, the average cost per tonne for incineration is £86, and the average cost for landfill (including Landfill Tax) is £107 [76]. The mass of a single-use face mask is assumed at 2.68 g, while the mass for the reusable counterpart is assumed at 14.4 g. Thus, the annual cost of discarding 365 single-use face masks would be £0.09, and the annual cost of disposing of 12 reusable masks would be under £0.02. The disposal cost for the reusable scenario is around 5.7 times cheaper.

The total annual cost (considering product price, washing and disposal) would be £95 for the single-use face mask scenario and £26 for the reusable option (rounding to the nearest pound) (Table 5). From a life cycle perspective, an annual provision of single-use masks for one UK citizen would cost around 3.7 times higher than the annual supply of reusable face masks. It is worth noting that the cost of washing reusable masks (122 washes) over a year is £1.22 and represents only 4% of the total annual cost for the reusable scenario (Fig. 3).

Considering a UK population of 67.8 million [68], the single-use scenario results in an annual cost of £6.4b, while the reusable option would cost £1.7b. If reusable face masks were to be made mandatory (and single-use masks restricted), this would represent annual savings of £4.7 billion for the UK economy. The cost analysis model assumes that most face masks (either disposable or reusable), or the raw materials used to manufacture them, are imported from abroad (e.g., China). Costs of finished products and raw materials are likely to spike due to high demand. As the manufacture of single-use masks typically requires specialised machinery, capital costs to begin their production in the UK would be higher than the local manufacturing of reusable masks. Reusable mask manufacture can be scaled up reasonably easily in the UK, providing a boost to the UK economy without impacting on the supply of single-use masks to the NHS. Countries such as Portugal [77] and France [78] have issued guidance for the manufacture of such masks, including a ‘stamp of quality’ in Portugal based on the Directorate-General of Health guidelines [77].

## Discussion and conclusions

**Figure 4:**
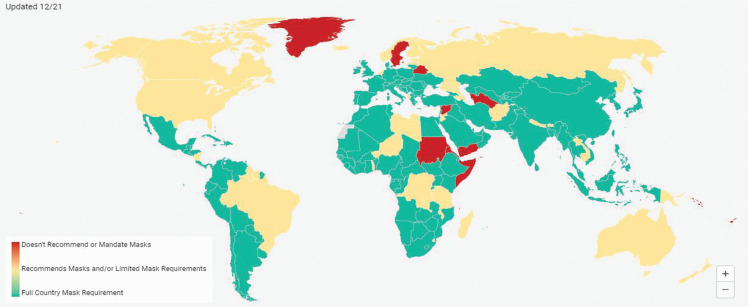
Mask requirements and usage by countries, obtained from Mask4All, latest update on 24 June 2021 [80]. Green indicates countries where they had countrywide mandatory mask use laws in place or had reached universal usage (mask use by >80% of the population) through recommendations from the government. Yellow indicates countries where mask use was mandated by law in parts of the country and recommended by government in other parts. Red indicates countries where no mask laws and no recommendations were given by the government.

Many governments around the world have introduced policies that recommend or mandate the wearing of masks to slow the spread of COVID-19 (Fig. 4). Mandatory use and enforcement varies globally [79]. While many countries have introduced laws to mandate mask use, countries such as China, India, Japan, South Korea and Taiwan have made more limited recommendations [80]. Around the world, policies also differ on whether children, or children of certain age, are exempt from mask wearing; the types of places and settings where masks are mandatory (i.e., all public or selected settings); and the fines that can be incurred for non-compliance [79–83]. Some countries, such as Italy and Mexico, provided single-use masks for the general public upon mandating their use [84]. Japan provided cloth masks without imposing mandatory use [84], while the Czech Republic and the UK advocated for the public to wear reusable masks and provided information on how to make them at home [85]. There is not yet enough data to determine which combination of mask policies is most effective in slowing the spread of infection. However, with billions of people now wearing masks globally, there are potentially high environmental and economic costs.

Countries such as the UK campaigned for the use of reusable masks mainly due to shortages of single-use masks in the healthcare sector. However, with better forward planning for the medical PPE supply, mask shortages may not be an issue in future. The analysis and discussion presented here can inform future mask recommendations and policy.

Over the last few decades due to decreased costs, the healthcare sector has adopted single-use masks over reusable ones in the belief they offer higher standards of hygiene both for the use phase and during waste management treatment (incineration). Availability and hygiene were also why the UK general public chose surgical masks and single-use respirators at the beginning of the COVID-19 pandemic.

Certified single-use masks have a higher filtration efficiency and fulfil higher performance criteria than most reusable masks. However, they do require new waste procedures and infrastructure for safe disposal in the form of incineration and have a higher environmental impact than reusable masks. In order to minimise the public health issues associated with the disposal of contaminated waste, and as littering of used surgical masks has been observed, local councils could install special disposal units for contaminated masks in public spaces.

As we have shown, there is growing scientific evidence that reusable masks can be made to satisfy high filtration performance criteria. From a public health perspective, what is less clear is how the level of mask filtration efficiency translates to reduced infection rates at a population level. For instance, there is anecdotal evidence of people reusing single-use masks, which may increase risk of infection, or washing them, which could compromise filtration efficiency. Similarly, if reusable masks are not laundered after every use, or laundered more than the recommended number of times, this too will challenge their filtration efficiency.

The filtration efficiency is not the only factor that determines the impact of mask wearing. The behaviour associated with mask wearing such as donning and doffing the mask, correct cleaning or disposal, and indirect impacts of mask wearing such as how it affects adherence to social distancing and hand washing, all indirectly affect the efficacy of mask wearing. We have noted that there are limited behavioural studies that differentiate and compare the impact of single-use or reusable masks on these behaviours. Extrapolating from related evidence, single-use masks may be chosen by citizens due to perceived increased hygiene and convenience of not having to wash them. By contrast, people may choose reusable masks due to the lower financial costs or the smaller environmental impact when compared with single-use masks. Further behavioural research is needed to fully understand whether our assumptions about these factors are reasonable, and also to identify barriers and enablers to wearing and cleaning reusable masks. For instance, a study comparing the use of single-use and reusable masks with the same level of standalone performance (i.e., equal filtration efficiency) by different communities will be necessary to understand whether the additional process of laundering masks affects the overall compliance and therefore effectiveness of mask use in slowing down the rate of pathogen transmission.

A simple yet plausible study could be carried out by utilising the large amount of data and engaging with participants through symptom monitoring Apps (e.g., ‘COVID Symptom Study app’ by King’s College London [86]). In addition to symptom monitoring, these platforms are also capturing mask use habit data and COVID-19 infection rates. Analysis of such data with follow-up interviews where members of the public answer specific questions on their practices related to mask use would be invaluable. For instance, interview questions could include: which type of masks (if any) they use; what individual (e.g., values, beliefs, attitudes and perceptions), social (e.g., culture and norms) and situational factors (e.g., context and cost) influence their mask use; what situations do they use them in; and, crucially, how often do they wash reusable masks? The findings of such a study would be valuable for understanding how mask-wearing behaviours impact infection rates and thus inform government policy.

A significant outcome of our analysis shows that choosing to wear reusable masks generates 85% less waste, has 3.5 times lower impact on climate change and incurs 3.7 times lower costs than single-use masks. These lower impacts depend on our assumptions, for example, a limited number of reusable masks used per person and the use of washing machines to regularly clean them. Even if these assumptions are lower than the actual number of reusable face masks purchased, for instance, the environmental significance of reusable masks is still clear. From this perspective, there is a strong argument that public policies should encourage wearing reusable masks to reduce our waste and carbon footprint.

From a cost perspective, our analysis demonstrates that there is presently a £70 per person difference between single-use and reusable masks (assuming one person uses one mask per day for a year). To prevent future shortages, governments may consider stockpiling single-use masks or ensuring sufficient local production chains, which would increase this per person cost difference, as storage and distribution costs would be necessary. Upfront costs of reusable masks may also increase as their performance levels increase. Furthermore, the actual number of reuses may be lower than we have assumed due to potential misplacement, unforeseen damage and even visual dislike, resulting in a higher number of masks being necessary. All of this would narrow the cost between single-use and reusable masks. Currently, our assumption of 12 reusable masks per person per year means that the price of these masks could increase up to £7 per mask before it matches the cost of single-use masks. An understanding of the actual number of masks, single-use and reusable, that are necessary to support an individual would inform a better cost comparison and a better environmental impact comparison.

An alternative policy to stockpiling is to promote resilience through local in-country production of masks. For single-use mask manufacture, it would be necessary to consider the potential import of raw materials since the UK does not currently manufacture non-woven fabrics on a large scale. Importing raw materials from Turkey rather than China would generate a lower environmental impact (see Appendix 3). Assessing the impact of producing reusable masks in the UK and/or other countries was not carried out but it is likely that if production is shifted from China to the UK, the overall environmental impact would reduce and remain lower than single-use masks. The UK would want to ensure that such agile manufacturing capacity is capable of scaling up quickly (in a matter of weeks). Hence a hybrid plan that combines stockpiling enough masks for the whole population coupled with a set of financial incentives for garment manufacturers to maintain the capability to retool factories for maskmaking may be the best compromise between cost and resilience.

## Figures and Tables

**Figure A1:**
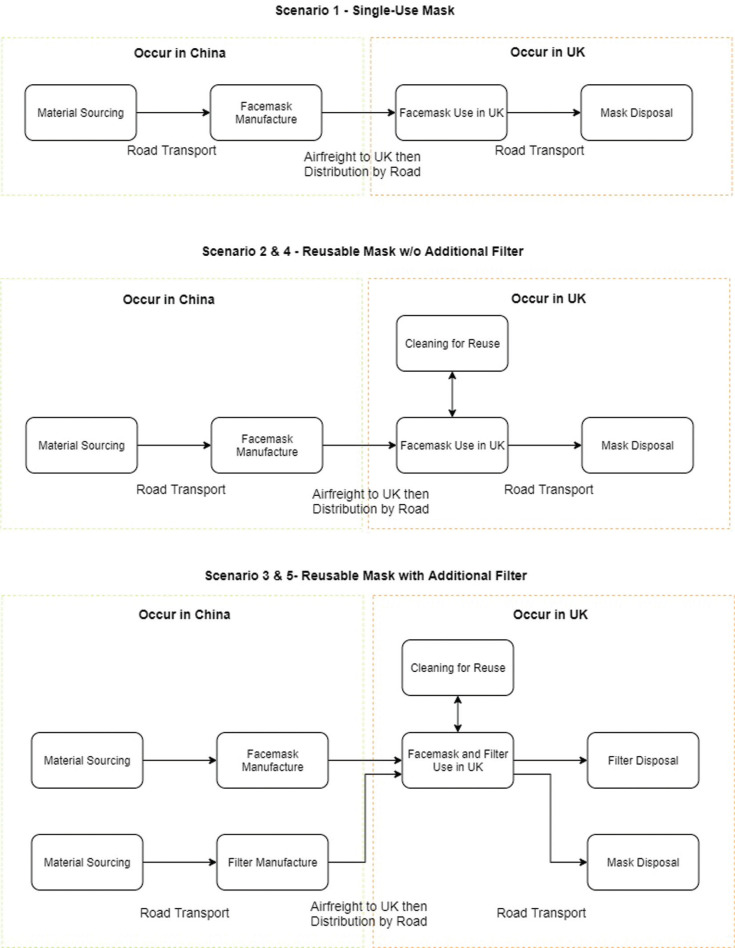
Cradle-to-grave system boundary for each face mask use scenario.

**Figure A2:**
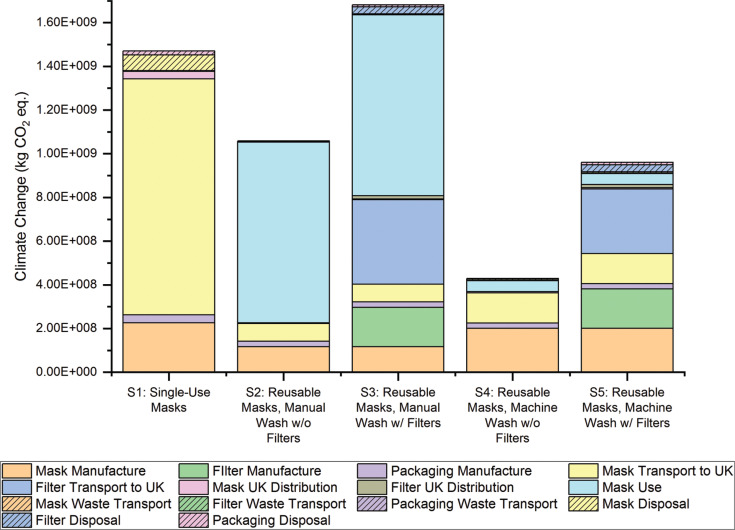
Climate change results generated for each scenario of face mask use.

**Figure A3:**
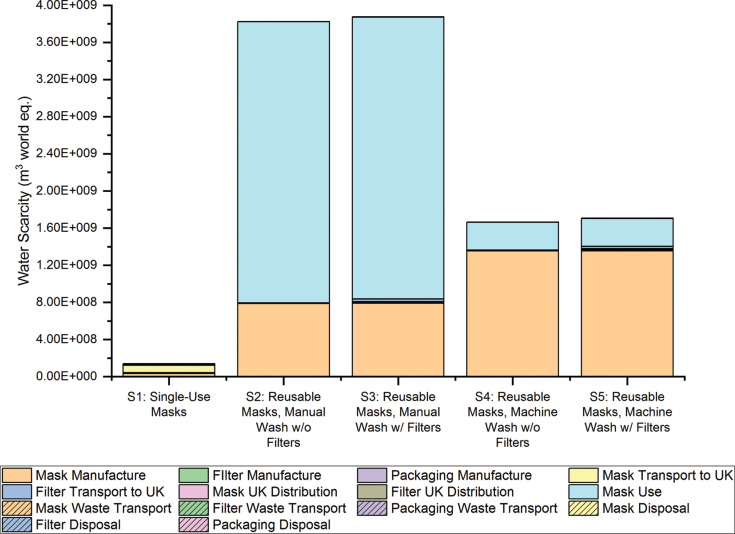
Water Scarcity results generated by each face mask scenario.

**Figure A4:**
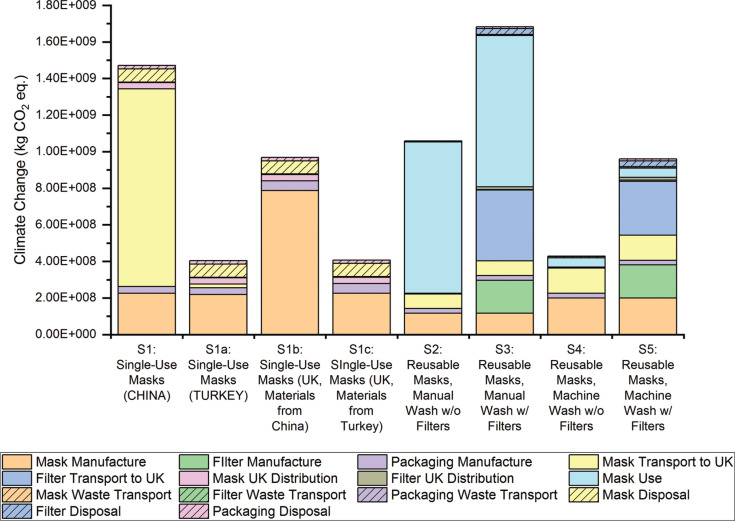
Climate Change results generated for each scenario of face mask use and single-use face mask supply.

## Data Availability

All data generated or analysed during this study are included in this published article (and its supplementary information files).

## Appendices

### Appendix 1: European Standards – Classification and Use Summary

**EN14683:2019 *‘Medical face masks – requirements and test methods’*** defines masks as medical devices when performance criteria, particularly breathability, microbial cleanliness and bacterial filtration efficiency (BFE) amongst other requirements are met. The standards outline the procedure for testing each criterion. For BFE, it requires masks to be exposed to *Staphylococcus aureus*, a bacterium up to 1 μm in diameter and quantifying the amount that are able to penetrate from the outer to the inner layer [8]. Medical grade masks are then classified into Type I and Type II based on their level of BFE. Type IIR have the same BFE as Type II but have additional requirements for breathability and splash resistance. The standard states ‘Type I medical face masks should only be used for patients and other persons to reduce the risk of spread of infections particularly in epidemic or pandemic situations. Type I masks are not intended for use by healthcare professionals in an operating room or in other medical settings with similar requirements’ [8]. Healthcare professionals hence require face masks meeting Type II and Type IIR standards.

The surgical mask is the widely used name for single-use medical face masks. Prior to the COVID-19 pandemic, reusable medical face masks were not introduced. Government decisions to recommend reusable face coverings for the general public induced some companies to produce reusable masks that satisfy Type I criteria to assure users of their products’ effectiveness. However, when the CEN further published the workshop agreement on community face coverings, companies begun conforming to the newer guidelines, which specify the requirements for masks intended for use in a community setting (below).

**EN149:2001 ‘*Respiratory protective devices – filtering half masks to protect against particles – Requirements, testing, marking*’** defines masks as personal protective equipment and referred respirators (sometimes filtering facepieces) when performance criteria, particularly (particle) leakage and breathability, amongst other requirements are met. The standard requires masks to fulfil two levels of leakage evaluation: the filtering material must meet filtration efficiency requirements and the overall mask must not exceed total inward leakage limits through the face seal, the filter and the exhalation valve (if applicable). Filtration efficiency and inward leakage are tested separately against aerosolised sodium chloride and paraffin oil size ranged between 0.02 and 2 μm (median average of 0.6 μm) [18]. Masks are then classified as FFP1, FFP2 and FFP3 based on the two performance criteria. Compliance to this standard means that they meet all the performance criteria that allows the user to be protected against solids, water-based aerosols and oil-based aerosols. The UK government recommends that healthcare workers are to use FFP3 classed respirators when caring for patients in situations where there is a high risk of bacterial and viral aerosols. FFP2 are currently allowed when FFP3 are unavailable. Due to the stricter requirements on inward leakage and the smaller particle size used for testing, respirators are often assumed to be more effective at filtering viruses than medical masks.

Note that not all respirators available for purchase are CE marked but may be certified according to similar standards established by other countries. Commonly referenced standards include the NIOSH:42CFR84 US standard [30], where masks are classified to N95 (95% filter efficiency), N99 (99% filter efficiency) and N100 (99.97% efficiency) [27]. But these masks are not oil resistant as are FFP masks. Similarly, GB2626:2019 Chinese standard classify respirators to KN95, KN99 and KN100, which have the same filtration efficiency as the US standard [28]. N95/KN95 and N99/KN99 are deemed close in equivalence to FFP2 and FFP3, respectively [30]. The modified UK government policies on face masks used within the healthcare sector allow non-CE marked single-use medical masks and respirators to be used provided that documentations of their performance meet UK and EU standards. This meant that masks certified, or in the process of being certified, under EU and/or other countries’ standards are allowed to be used once approved by the HSE [29].

Conventionally, single-use respirators are preferred, particularly in the healthcare setting, due to hygiene requirements. Reusable variations are available due to their wide use in other sectors such as the construction industry where high levels of aerosols are present. For a respirator to be labelled reusable, it must pass clogging evaluations and maintain performance criteria for more than one shift. Traditionally, reusable respirators are often labelled to be sufficient for use for up to three shifts (assuming 8 hours per shift with wipe cleaning between uses) and recommends discarding when breathability reduces due to clogging. Due to increased needs for higher reusability, further reusable respirators have emerged claiming effectiveness for longer periods. As shown in Table 1, reusable respirators are familiar in their construction to single-use respirators but are usually composed of additional components that prolong the masks’ integrity.

Cambridge Mask and Respro® claim that their products are as effective as standard single-use masks, if used correctly. Cambridge Mask, which produces respirators made with UK military-grade filtration technology, claims their masks are effective for 340 hours [25]. The masks are claimed to filter out dust and pollution particles, categorised as particulate matter (PM), such as PM10 (10 μm), PM2.5 (2.5 μm) and PM0.3 (0.3 μm), with 95% efficiency, using a unique triple-layer filtration system. Alternatively, Respro® offers a number of general use respiratory masks with the interchangeable combination filter, claimed to be suitable for airborne viruses, that should be replaced every 69 hours [87].

**CWA 17553:2020 *‘Community face coverings – Guide minimum requirements, methods of testing and use’*** sets out guidance for mask performance, both single-use and reusable, and their use within the community. Unlike EU standards, CWAs involve no obligations; they are agreements that are open to participation. Thus, this workshop agreement acts as a set of recommendations for mask manufacturing companies to follow. However, compliance is desirable. As EU standards previously discussed, masks must meet breathability, filtration efficiency and other criteria to be compliant and be deemed a community face covering. It recommends the testing parameters for each performance criteria, in particular for filtration efficiency. It includes examples of the solid and liquid particles that masks should be tested against. Following this, masks are categorised into two levels of performance based on their filtration efficiency of particles sized approximately 3 μm [23]. For masks that aim to be labelled reusable, filtration efficiency must also be maintained through a minimum of five cleaning cycles, and the number of cleaning cycles for which the producer states, at 60°C [23]. In addition, recommendations for effective use of masks, including putting on and removing masks, social distancing and hand-washing procedures are also provided with the workshop agreement.

CWA17553:2020 was published in June 2020 after recommendation by many European governments to mandate the wearing of masks in public. As it was advised to use masks alternative to those required by healthcare professionals, with lower infection risks in community settings as one of the reasonings, this workshop agreement outlines mask performance recommendations for lower risk settings. Hence, the chosen particle size for test is set to ∼3 μm, larger than the test sizes for medical masks (∼1 μm) and respirators (∼0.6 μm). In addition, as the general advice is to use reusable masks, due to waste considerations, community face coverings emerging on the market are reusable.

**EN140:1999 *‘Respiratory protective devices – Half masks and quarter masks – Requirements, testing, marking’*** defines masks as facepieces that can provide adequate sealing around the face, particularly the mouth and nose (and chin for half masks), of the user from the immediate environment. This standard does not require masks to carry out particle filtration but requires them to fulfil breathability, inward leakage and carbon dioxide inhalation performance criteria. If the facepiece is intended to be used as a filtering device, then performance criteria must be met when the intended filter(s) are simulated. The inward leakage of no greater than 2% must be met under two test conditions, after 24-hour mask storage in dry atmosphere of 70°C and 24-hour storage in −30°C and with aerosolised sodium chloride (particles sized between 0.2 and 2 μm (median 0.6 μm) and/or sulfur hexafluoride [20].

The standard states that inhalation valves are not required, although preferred, and require a minimum of one exhalation valve (or a similar apparatus). It states that if the facepiece is intended to be used with filters then an inhalation valve is mandatory, either integral to the facepiece or the filter.

**EN143:2000 *‘Respiratory protective devices – Particle filters – Requirements, testing, marking’*** certifies filters to adequately prevent the penetration of liquid, solid and oil particles and therefore adequate for use as components in facepieces that are not constructed of filtering materials. Filters must fulfil breathability and particle penetration (filtration efficiency) amongst other criteria. The performance tests and classification are similar to EN149:2001. For filtration efficiency, filters must be tested, separately, against aerosolised sodium chloride and paraffin oil sized ranged between 0.02 and 2 μm (median average of 0.6 μm) [19]. They are then classified into P1, P2 and P3, with P3 requiring a higher filtration efficiency than FFP3 respirators.

The standard mandates the testing of filtration efficiency twice with a 24-hour storage between the tests. This is to calculate the average efficiency and to determine whether the filter can be classed as reusable. For filters to be labelled reusable they must pass clogging evaluations and maintain performance criteria for more than one shift. Like traditional reusable respirators, reusable filters are often labelled to be sufficient for use for up to three shifts (assuming eight hours per shift) and are recommended to be discarded when breathability reduces due to clogging.

**EN1827:1999 *‘Respiratory protective devices – Half masks without inhalation valves and with separable filters to protect against gases or gases and particles or particles only – Requirements, testing, marking’*.** This EU standard defines masks as reusable facepieces, intended to be used with single-shift, separable and replaceable filters, and provides adequate sealing around the face, particularly the mouth, nose and chin, against gases and/or particles within the immediate environment. Performance criteria are mostly specified for the complete device, this means the combination of the facepiece and filter(s) are required to be tested together. This includes breathability, clogging and practical performance criteria. Inward leakage testing under this standard indicates the leakage through the face seal and asks for facepieces to be tested with FM P3 class filters fitted or with clean air emulating from the facepiece [21].

The standard outlines the performance criteria for both gas filtering and particle filtering by the filter and tests must be carried out according to the filter’s intended use. Filters are classified FM followed by their gas filtration performance and/or particle filtration performance. Particle filtration is classified into P1, P2 and P3 as set out in EN143:2000 (above). As this paper concerns particle filtration, we will not summarise the requirement for gas filtration. As a whole, both EN140:1999 and EN1827:1999 facepieces with separable filters are used as reusable respirators but are not commonly used to prevent the spread of pandemic. Their performance with particle filters are equal and higher than EN149 respirators. Their low usage can be due to limited awareness of these masks and/or upfront costs.

### Appendix 2: Recommendations to Prevent Pathogen Transmission

One behavioural aspect of using face masks when in public is that they help prevent transmission indirectly by preventing touching of the face, particularly as this is when people are at increased risk of touching a contaminated surface and then touching a mucous membrane (i.e., nose, eyes and mouth). As summarised in Table A1, avoiding touching the nose, eyes and mouth is key to preventing the transmission of COVID-19 among the general population. Studies have shown that individuals touch their faces an average of 23 times per hour, 44% of which involves touching a mucous membrane [88]. Of the mucous membranes touched, the most common was the mouth, followed by the nose and eyes. Masks may be able to prevent transmission of the disease by acting as a physical barrier against mouth and nose touching when in public and for those most at risk of coming into contact with an infectious person. However, Casanova et al. have reported that, depending on the protocols taken for hygiene, the removal of PPE could result in virus transfer to hands and clothing [89]. Therefore, it is essential that users wash their hands and decontaminate their clothing.

**Table A1.** Recommended behaviours to prevent transmission of COVID-19 among the general population (taken from [55]).

Categories of recommended behaviours	Enabling behaviours
1. Maintain hygiene	
a. Cleaning hands	• Ensure ready access to soap and water or alcohol-based (60%+) sanitiser at all times. • Carry moisturiser if you are concerned about dry hands. • Learn how to wash hands effectively for 20 seconds, soaping the backs of your hands, between your fingers and under your nails. • Learn when to wash hands and use clearly specified ‘if-then’ plans to carry this out, for example, ‘If I touch a potentially contaminated surface, I will wash my hands as soon as possible afterwards’.
b. Using and disposing of tissues	• Make sure you always have clean tissues available. • Identify places to dispose of tissues immediately or as soon as possible. • Train yourself to cough or sneeze into tissues (or crook of elbow if not available), *not your hands*.
c. Cleaning surfaces	• Watch out for surfaces that could be contaminated. • Use household disinfectant to wipe at-risk surfaces.
2. Avoid touching	
a. Avoiding touching nose, mouth and eyes	• Make sure to keep hands below shoulder level except when, for example, hair-brushing. • When acceptable, ask for and give feedback when you or others are touching nose/mouth/eyes.
b. Avoiding close contact greetings	• Develop and use alternative greetings, for example, elbow-bumping, head-bowing. • Explain why you are not engaging in close contact greeting to make it normal and acceptable.
c. Avoid touching surfaces at risk of contamination	• Develop strategies for avoiding commonly touched surfaces where possible, for example, door handles. • Avoid handling other people’s personal objects, for example, mobile phones.
3. Social distancing	
a. Avoiding crowds	• Plan work, travel or recreational activities that do not involve physical social gatherings, for example, online social games and events. • Develop explanations to give to people as to why you are avoiding social gatherings.
b. Maintaining personal distance	• Avoid standing or sitting close to people who are showings signs of infection.
c. Isolating (if advised to do so)	• Plan activities to minimise boredom and frustration in case of possible isolation. • Plan for practicalities of maintaining everyday life, for example, medicines, food, communications. • Plan for financial and social support during possible isolation.

**Table A2.** Summary of scenarios compared in the comparative study.

Scenario number	Mask type	Mask use per day	Reuse model	Mask treatment	Number of masks per person per year	Addition filters	Number of filters per person per year	Filter treatment
1	Single-use surgical mask	1	N/A	Disposed at the end of day	365	No	0	N/A
2	Reusable cotton mask	1	Two masks in rotation	Manual washing, 50 washes	7	No	0	N/A
3	Reusable cotton mask	1	Two masks in rotation	Manual washing, 50 washes	7	Yes	365	Disposed at the end of day
4	Reusable cotton mask	1	Four masks in rotation	Machine washing, 30 washes	12	No	0	N/A
5	Reusable cotton mask	1	Four masks in rotation	Machine washing, 30 washes	12	Yes	365	Disposed at the end of day

### Appendix 3: An LCA comparison between single-use and reusable face masks in the UK

#### Background

Many countries have introduced the mandatory use of face masks as a non-clinical intervention to reduce the spread of SAR-Cov-2; this includes the UK. However, a rise in single-use face mask littering has been observed, leading to environmental concerns over their use. An MFA analysis carried out (to complement this paper) suggests that if single-use face masks were widely used by UK citizens, then this will amount to 48 kt of plastic (66 kt total waste) that would need to be disposed of annually. Although the literature states that, in a clinical setting, single-use face masks are currently more effective than reusable cloth (uncertified) ones, some experts have suggested that, for general use, reusable masks are just as adequate at preventing transmission when used correctly [90,91]. Reusable face masks can potentially reduce the amount of resultant waste, but, due to differences in material composition and the cleaning processes necessary for reusable face masks, a trade-off in environmental impacts may arise. In addition, some reusable face masks can be complemented with single-use filters to offer greater air filtration. This may reduce the resultant waste from using single-use face masks, but, equally, a high amount of waste for disposal can be foreseen. This study aims to understand the environmental impacts of both single-use and reusable face masks if they are nationally adopted in the UK.

#### Goal

To compare the environmental impacts of using single-use (surgical masks) and reusable cloth masks nationally to prevent the transmission of infection in the UK.

#### Scope

Five scenarios for the public use of face masks were analysed in this comparative study:

The **FU** employed for the analysis is **1 year of mask use by the UK population**, assuming one face mask used per person per day. The number of face masks and filters required to support use in this period was calculated according to the UK’s population (Table A3). This study acknowledges that the use of one mask per person per day may not be reflective of actual mask use during a pandemic in the UK where members of society can be exempt or are practising shielding. However, there are also members of society who are required to attend work and therefore require the use the face masks. Under recommendation by the PHE and the WHO, the maximum use duration of surgical masks is 6 hours (ranging 2–6 hours) and should be discarded once moist or wet [66]. Another study has stated that surgical masks are not effective after 4 hours [67]. Hence, depending on an individual’s responsibilities and lifestyle, the number of masks per day would differ and can balance those who use fewer than one mask per day.

**Table A3.** Number of face masks and filters required to support face mask usage in the UK for 1 year.

Scenario number	Mask type	Functional unit/time frame	Number of masks	Number of filters
1	Single-use	1 year	24.7 billion	N/A
2	Reusable	1 year	475 million	N/A
3	Reusable	1 year	475 million	24.7 billion
4	Reusable	1 year	814 million	N/A
5	Reusable	1 year	814 million	24.7 billion

The assumed UK population was 67.8 million [68].

**Table A4.** Material of construction and mass used to model each product.

Product/component	Material	Area (m^2^)	Length (m)	Mass (g)	Source/reference
(S1) Single-use mask					
Layer 1	PP (non-woven)	0.029	–	0.638	95 mask (2020) and Thomasnet (2020) provided the components and dimensions of a surgical mask
Layer 2	Cellulosic fabric	0.029	–	0.725	
Layer 3	PP (non-woven)	0.029	–	0.638	
Nose wire	HDPE	–	0.098	0.231	
Ear loops	Polyetherimide (elastic material)	–	0.185 (each)	0.444	
Total				2.68	
(S2) Reusable mask					
Layer 1	Cotton fabric	0.039	–	6.98	CDC [93] provided the dimensions of fabric required
Layer 2	N/A	–	–	–	
Layer 3	Cotton fabric	0.039	–	6.98	
Nose wire	N/A	–	–	–	
Ear loops	Polyetherimide (elastic material)	–	0.185	0.444	
Total				14.4	
(S3) Single-use filters					
Layer 1	PP (non-woven)	0.0096	–	0.211	Product dimensions taken from product specification from Amazon.co.uk. Materials assumed similar to single-use masks
Layer 2	Cellulosic fabric	0.0096	–	0.241	
Layer 3	Carbon filter (activated carbon)	0.0096	–	0.288	
Layer 4	Cellulosic fabric	0.0096	–	0.241	
Layer 5	PP (non-woven)	0.0096		0.211	
Total				1.19	

**Table A5.** Electricity assumptions for the manufacture of masks and filters.

Product/component	Electricity consumption (kWh/1000 mask)	Reference values	Assumption/reference
Single-use mask			
Mask body forming	0.556	4 kW, 110–160 pcs/min	Reference values were taken from Testex (no date) website on surgical mask production line. It was assumed the thoroughput of masks was 120 pc/min (240 pc/min of ear loops)
Ear loops cutting	0.694	0.5 kW, 120–240 pcs/min	
Ultrasonic welding	0.167	1.2 kW	
Total	0.792		
(Total per FU)	(19.6 GWh)		
Reusable mask			
Laying, cutting and sewing	34.2	2.38 kWh/kg	[95]
Total	34.2		
(Total per FU)	(4.64 GWh)		
Single-use filters			
Filter body forming	0.556	4 kW, 110–160 pcs/min	Assumed similar production to single-use masks (above)
Total	0.556		
(Total per FU)	(13.7 GWh)			

FU: functional unit.

Furthermore, the difference in environmental impact between the different scenarios is relative. A scaling factor can be applied to the environmental impact results to reflect the actual impacts if the average mask use quantity per person changes. Reusable masks were modelled as cotton if used in rotation: if an individual has two masks, it was assumed that only manual washing is possible due to the necessity of frequent washes. With four masks, it was assumed that they could be bulk washed with normal laundry. It was assumed further that masks can withstand 30 washes in the washing machine [40] whilst 50 washes by manual washing as hand washing of clothes is a typical method used to preserve clothing items.

A cradle-to-grave study approach was used for this comparison. The scope of the study included the material sourcing of the face masks, transport to the manufacture facility, the manufacture of face masks, transport to the UK, face mask distribution nationwide, and face mask use and final disposal (Fig. A1).

#### Manufacturing assumptions

It was assumed that the face masks (both single-use and reusable) and filters were manufactured in China before being transported by airfreight to the UK. The materials and energy assumed to be required for the major manufacturing process of face masks and filters are summarised in Tables A4 and A5. For all five scenarios, the arising waste and treatment of waste from manufacturing was not modelled due to limited data. Their impact was also assumed to be relative amongst the scenarios. The emissions associated with the life cycle of factory machines were also not modelled. This was because installed equipment is assumed to have a long lifespan, 30 years on average [92]. The emissions and environmental impacts associated with the fabrication and decommission of equipment would be allocated proportionally over their lifespan, and was, therefore, assumed to be negligible.

#### Packaging assumptions

Packaging configurations were assumed based on product specifications shown on retailers’ websites [94,96,97]. Table A6 details the assumptions made in calculating the packaging mass of each packaging component.

**Table A6.** Packaging assumptions for each scenario.

	Packaging configuration	Component/material	Component mass (kg)	Total mass per FU (kt)	Assumptions/reference
S1 – single-use face mask	50 pcs/box 40 boxes/carton (2000 pcs/carton)	Box – cardboard Carton – cardboard	0.0535 2.50	1060 30.9	LANS Grupo [96] provided dimensions and mass of each packaging component
S2 – reusable face masks, manually washed (two masks per person)	Individually wrapped 1500 pcs/carton	Wrap – LDPE Carton – cardboard	0.00335 2.50	0.454 0.226	0.09 m^2^ surface area and 40 μm thickness of LDPE sheet was assumed to provide the mass per component Assumed same size carton used, number of pcs per carton was calculated based on face mask surface area differences
S3 – reusable face masks, manually washed, with single-use filters (two masks per person)	Masks individually wrapped 1500 pcs/carton Filter wraps in packs of 10 6000 pcs/carton	Wrap – LDPE Carton – cardboard Wrap – LDPE Carton – cardboard	0.00335 2.50 0.00186 2.50	0.454 0.226 4.6 10.3	0.05 m^2^ surface area and 40 μm thickness of LDPE sheet was assumed to provide the mass per component Assumed same size carton used, number of pcs per carton was calculated based on face mask surface area differences
S4 – reusable face masks, machine washed (4 masks per person)	Individually wrapped 1500 pcs/carton	Wrap – LDPE Carton – cardboard	0.00335 2.50	0.454 0.226	(As Scenario 2)
S5 – reusable face masks, machine washed, with single-use filters (four masks per person)	Masks individually wrapped 1500 pcs/carton Filter wraps in packs of 10 6000 pcs/carton	Wrap – LDPE Carton – cardboard Wrap – LDPE Carton – cardboard	0.00335 2.50 0.00186 2.50	0.908 0.452 4.6 10.3	(As Scenario 3)

#### Transport assumptions

Both face mask types were assumed to have been manufactured in China before being distributed in the UK, with transport assumptions shown in Table A7.

**Table A7.** Transport assumptions for masks and filters for all scenarios.

	Mode of transport	Distance (km)	Notes
Materials to manufacturing facility and facility to terminal	Truck	100	Assumed materials sourced locally
China to UK	Airfreight	7800	[98]
Mask distribution	Truck	1000	Assumed distribution start from one UK terminal
Mask and filters to disposal sites	Truck	100	Assumed local authority collection for disposal

#### Reuse assumptions

MacIntyre et al. have recommended using at least two face masks in rotation to allow for adequate cleaning and drying before use. It is acknowledged that the number of reusable masks used in rotation per person depends on personal preference and economic feasibility. Hence, scenarios where two and four face masks are employed per person were both modelled. Due to the frequency of washing required it was assumed that, with two face masks, manual washing is necessary. With four masks, it was assumed that households could bulk wash their face masks with the usual laundry, and therefore machine washing is possible (explanation of assumptions below).

The International Scientific Forum on Home Hygiene has published a report on the infection risks associated with clothing [47]. It states that laundering processes will eliminate contamination from fabric and bedlinen materials. For this study, an average household soap/detergent was assumed sufficient for cleaning face masks.

**Manual washing (Scenarios 2 and 3):** The study assumed that each face mask is washed every two days, due to being used in rotation. Hence, each reusable face mask is modelled to be washed 183 times per FU (1-year timeframe). Because frequent washing would be required, manual washing of face mask was assumed. Ariel’s guide on hand-washing recommends using a teaspoon (approximately 6 ml [6.24 g]) of liquid detergent in a tub of slightly warm water. Once the garment has been cleaned with the mixture, it should be rinsed in a tub of detergent-free water [99]. The tub volume was not mentioned, but this was assumed to be a 5 l-washing bowl filled to 3 l level.

The Office for National Statistics states that an average household comprises 2.4 people [100]. Therefore, it was assumed that 2.4 masks could be washed together. Hence, each mask requires 2.6 g of detergent and 2.5 l of water per wash. It was assumed that hot water from household taps is typically heated up to 60°C by gas boilers [101]. The total requirements for mask cleaning are shown in Table A8.

**Table A8.** Requirements for the manual washing of face masks for Scenarios 2 and 3.

Cleaning components	Per mask per wash	Per person per year (183 washes)	Total per FU
Soap	2.6 g	476 g	62.1 kt
Water	2.5 l	458 l	6.21 × 10^10^ l
Steam	407 kJ (Q = mcdT = 2.5 kg × 4.186 kJ/kg × (60°C –21°C))	74.5 MJ	10.1 PJ

Note: That users may opt to wash their masks under running water. As the average tap water flow rate within UK is between 4 and 6 l/min [102], and depending on the soap, masks may need longer rinsing time than hand washing. We believe that washing masks in tubs of water is more sustainable than washing under running water.

**Machine washing (Scenarios 4 and 5):** This study assumed that, within an average household comprising 2.4 people, there would be sufficient laundry for a full machine wash every 3 days (if garments from each household member were pooled). One wash every 3 days means that each face mask is washed 122 times in 1 year (FU). Walser et al. evaluated the environmental impact of t-shirts, with consideration for the ‘low’, ‘medium’ and ‘high’ environmental awareness of their wearers, which influences the choice of washing machine category, the quantity of detergent used, and the temperature of the wash [26]. Acknowledging that the ability to own a highly efficient washing machine is also dependent on household income, it was assumed that the ‘medium’ scenario is more probable for the UK public. Hence, this study used the parameters assumed by Walser et al. in their ‘medium’ scenario (Table A9), a 40°C full-load wash, to allocate the amount of cleaning resources required to clean each face mask [26].

**Table A9.** Requirements for the machine-washing of face masks for Scenarios 4 and 5.

Cleaning components	Per machine wash of 6 kg load [26]	Per mask per wash	Per person per year (122 washes)	Total per FU
Soap	67.5 g	0.162 g	19.7 g	5.34 kt
Water	49 l	0.117 l	14.3 l	3.88 × 10^9^ l
Electricity	0.66 kWh	1.58 Wh	0.192 kWh	52.2 GWh

Note: the authors of this study acknowledge that the temperature advised is 60°C, which means the electricity consumption per load should be modelled as 1 kWh [26]. As the contribution of electricity due to machine washing in Scenario 4 and 5 is low (<10%) for all impact categories analysed, increasing electricity consumption ×1.5 will increase the overall value for each impact category by a maximum of 5%. With this in mind, the LCA model was not revised as the ranking amongst the scenarios will not change.

#### Disposal assumptions

All waste arising from the use of face masks was modelled for disposal through landfill and/or incineration: 43% landfill, 41% incineration with energy recovery and 16% incineration only. This was based on UK statistics on waste supplied by the Department for Environment, Food & Rural Affairs [Defra] [103]. Landfill and incineration were chosen as the disposal methods, because these are the typical waste destinations for household waste. Single-use face masks and filters are not currently recycled, while textiles are currently unlikely to be recycled. Although packaging can be recycled, plastic film packing, modelled as wrapping for reusable and single-use filters, is not conventionally recycled. Cardboard is widely recycled; however, this was not modelled due to insufficient data from GaBi [104] and EcoInvent databases [105].

For Scenarios 2–5, all face masks were modelled for disposal after 1 year of use. There is no data available on how long each reusable face mask can last; data is required to understand the usability of face masks after frequent washes. It was assumed that the life of each face mask would be similar. In Scenarios 2 and 3, the face masks are washed more frequently than in Scenarios 4 and 5; however, manual washing is typically recommended for delicate garments, because it is gentler on the fabric.

#### Results

The comparative study was modelled on GaBi Software [106], the LCIA method used to assess each scenario’s environmental impact was the EF 3.0 methodology [70]. Both the life cycle inventory (LCI) analysis and LCIA were carried out and compared across the different scenarios. The LCI analysis showed that the use of reusable face masks significantly reduces the amount of waste entering the general waste stream (Table A10). Due to packaging requirements, the total waste accumulated from using single-use face masks nationally amounts to 124,000 tonnes. If single-use filters are used in addition to reusable face masks, then the amount of waste is 50% less than using single-use face masks. There is >85% reduction in waste if only reusable face masks are used.

**Table A10.** Waste arising (thousand tonnes per FU) due to face mask use in the UK for 1 year.

	S1 – single-use masks	S2 – reusable masks, manually washed, w/o filter	S3 – reusable masks, manually washed, w/ filters	S4 – reusable masks, machine washed, w/o filter	S5 – reusable masks, machine washed, w/ filters
Waste arising per FU (kt)					
Masks	66.2	6.83	6.83	11.7	11.7
Filters			29.5		29.5
Packaging	57.4	2.38	17.3	4.08	19.0
**Total**	**123.6**	**9.21**	**53.6**	**15.78**	**60.2**

FU, functional unit.

A summary of environmental impact results is presented in Table A11. The results show that Scenario 4, in which four face masks are employed per person (without single-use filters) and are machine-washed, generated the lowest environmental impact in all impact categories, except the impact associated with water usage. The results also showed that when reusable face masks are employed without the additional use of single-use filters, whether they are washed manually or by machine, a lower environmental impact is generated overall. The use of single-use filters with reusable face masks is observed to be environmentally beneficial when compared to single-use face masks, if the masks are machine washed (Scenario 5).

**Table A11.** Overall environmental impact results for each face mask scenario.

	Scenario 1 – single-use masks	Scenario 2 – reusable masks (manual washing)	Scenario 3 – reusable mask with single-use filters	Scenario 4 – reusable masks (machine washing)	Scenario 5 – reusable masks with single-use filters (machine washing)
EF 3.0 Acidification terrestrial and freshwater [mole of H+ eq.]	6.27E+06	2.98E+06	5.12E+06	2.65E+06	4.62E+06
EF 3.0 Cancer human health effects [CTUh]	6.10E-01	5.10E-1	7.22E-01	3.35E-01	5.31E-01
EF 3.0 Climate change [kg CO_2_ eq.]	1.47E+09	1.04E+09	1.61E+09	4.10E+08	9.64E+08
EF 3.0 Ecotoxicity freshwater [CTUe]	1.56E+10	1.18E+10	1.71E+10	7.32E+09	1.23E+10
EF 3.0 Eutrophication freshwater [kg P eq.]	4.94E+04	6.72E+04	8.30E+04	3.43E+04	4.95E+04
EF 3.0 Ionising radiation - human health [kBq U235 eq.]	8.05E+07	2.27E+07	5.53E+07	2.37E+07	5.32E+07
EF 3.0 Land use [Pt]	4.68E+09	1.09E+10	1.28E+10	7.56E+09	9.34E+09
EF 3.0 Non-cancer human health effects [CTUh]	7.63E+00	1.49E+01	1.83E+01	1.55E+01	1.88E+01
EF 3.0 Ozone depletion [kg CFC-11 eq.]	2.60E+02	3.49E+01	1.15E+02	3.92E+01	1.12E+02
EF 3.0 Photochemical ozone formation – human health [kg NMVOC eq.]	6.10E+06	1.81E+06	3.89E+06	1.46E+06	3.38E+06
EF 3.0 Resource use, energy carriers [MJ]	2.15E+10	1.49E+10	2.35E+10	5.37E+09	1.34E+10
EF 3.0 Resource use, mineral and metals [kg Sb eq.]	4.87E+02	8.67E+02	1.11E+03	4.75E+02	7.11E+02
EF 3.0 Respiratory inorganics [Disease incidences]	3.23E+01	4.57E+01	5.73E+01	4.54E+01	5.65E+01
EF 3.0 Water scarcity [m^3^ world equiv.]	1.40E+08	3.82E+09	3.87E+09	1.66E+09	1.71E+09

Green indicates the lowest results generated; red indicates the highest results generated.

Figure A2 highlights the hot-spot analysis carried out on the Climate Change results generated by each scenario. It shows that the transportation of single-use face masks (Scenario 1) contributed most to this impact category. This is attributed to the large number of face masks required, and, therefore, an increased level of transportation is necessary, when compared to reusable face masks, to supply to the whole UK population for a year. The contribution of Mask Manufacture is also higher in Scenario 1, due to the higher quantity of masks required. For Scenarios 2 and 3, the highest contributor to Climate Change is the cleaning of masks for reuse; the thermal energy required to supply hot tap water represents over 70% of Scenario 2’s impact. In Scenario 4, it generated the lowest impact overall, even though a higher number of masks is required than in Scenarios 2 and 3. This suggests that having a higher number of masks in rotation, to allow for machine washing (Scenarios 4 and 5), is more environmentally beneficial than manual washing (Scenarios 2 and 3).

The results show that the use of reusable face masks can be environmentally beneficial when compared to using single-use face masks (Table A9). However, all reusable face mask scenarios are associated with substantial amounts of water usage. Figure A4 illustrates the processes that contribute to water scarcity. Reusable face mask manufacture (Scenarios 2–5) contributed highly to this impact category, when compared to the manufacture of single-use face masks. This is attributed to the high water requirements of the textile industry for producing cotton fabric. The most significant impact on water scarcity is manual washing of face masks within the ‘Mask Use’ life cycle stage (Scenarios 2 and 3). This caused the value generated by Scenario 2 and Scenario 3 to be two orders of magnitude larger than Scenario 1. (Note that ‘Mask Use’ considers the washing process and all other processes associated with washing, including energy generation for heat and production of soap.)

#### Further study on single-use mask manufacture

The hot-spot analysis showed that for Scenario 1 (single-use face mask), the largest contributor to the environmental impact categories was mask transport. This suggests that if the manufacture of single-use face masks is relocated, then the overall impact associated with single-use face masks will be reduced. China was modelled as the manufacturing location for single-use face masks because it is the biggest supplier of this product. The study therefore presents a realistic representation of the environmental impacts if single-use face masks are to be employed for everyday use in the UK. However, to combat the shortage of single-use face mask supply, textile companies have begun to convert their production lines to enable the production of face masks. This means the supply chain of face masks may change in the future.

Scenario 1 was further modelled to stipulate future supplies of single-use face masks (Table A12). Two manufacturing locations were modelled: Turkey (Scenario S1a), and the UK (Scenarios S1b and S1c). Turkey was assumed to be a viable location for the production of single-use face masks because it is the second biggest supplier of textiles after China [107] and one of the biggest producers of non-woven products in Europe [108]. Furthermore, Triton Market Research [109] showed that major companies that produce polypropylene (PP) non-woven products include those manufactured in Turkey. Hence, the material required to produce face masks in Turkey was assumed to have been locally sourced.

**Table A12.** Further modelling of Scenario 1 with changes made to production location and hence supply distances.

Scenarios	Materials sourcing	Mode of transport to plant/distance	Model of transport to UK terminal/distance	Justifications/reference
S1a – single-use masks manufactured in Turkey	For mask and packaging: Turkey	Truck/100 km	Truck/3500 km	Distance from Turkey to UK, assumed production plant situated in Istanbul and delivered to Dover (∼3000 km [110]). Additional 500 km was added to reflect transport to a distribution point
S1b – single-use masks manufactured in UK [1]	For masks: China For packaging: UK	Airfreight/7800 km Truck/500 km Truck/100 km	N/A	Transport distance from China assumed as Table A1. Additional 500 km was added to reflect transport to a distribution point
S1c – single-use masks manufactured in UK [2]	For masks: Turkey For packaging: UK	Truck/3500 km Truck/100 km	N/A	Transport distance from Turkey assumed as above

According to market data from the Organisation for Economic Co-operation and Development (OECD, [71]) and Edana [108], the UK is one of the main importers of non-woven textiles. Hence, it is deemed likely that the UK will need to import these materials in order to manufacture single-use face masks. Thus, if face mask production is relocated to the UK, and therefore the emissions associated with importing the product are eliminated, there will be emissions associated with importing raw materials. As China and Turkey are the largest suppliers of textiles [107], it was assumed that the UK will import the materials necessary for face mask production from either country. Scenarios S1b and S1c were modelled to explore the potential range of environmental impacts associated with producing in the UK (Table A11).

For Scenarios S1a–S1c, it was assumed that packaging for face masks is manufactured locally. This is because cardboard is largely produced in both Turkey and the UK [111].

#### Further results

A summary of the environmental impact results generated from modelling single-use face mask production (from Scenario 1) in Turkey and the UK are highlighted in Table A13. The results were compared to the impacts generated by Scenario 1, where single-use face masks are manufactured in China, and Scenario 4, where reusable face masks (manufactured in China) are used in rotation and machine washed. Results show that, by relocating single-use face mask production from China to Turkey and the UK, the environmental impacts will reduce by 39.4%, if the UK manufactures face masks but imports materials from China. In addition, over 81% reduction can be generated if materials are sourced from Turkey and manufactured in either Turkey or the UK. Scenario 1a (manufacturing in Turkey) becomes the most environmentally preferred, as it generated the lowest impact towards most environmental impact categories including Climate Change and Resource Use.

**Table A13.** Overall environmental impact results for the new single-use face masks supply scenarios compared to Scenario 4 (use of four reusable masks in rotation and machine-washed).

	S1 – single-use masks, manufactured China	S4 – reusable masks, machine washed, manufactured in China	S1a – single-use masks, manufactured in Turkey	S1b – single-use masks, manufactured in UK (materials from China)	S1c – single-use masks, manufactured in UK (materials from Turkey)
EF 3.0 Acidification terrestrial and freshwater [mole of H+ eq.]	6.27E+06	2.65E+06	1.18E+06	3.80E+06	1.02E+06
EF 3.0 Cancer human health effects [CTUh]	6.10E-01	3.35E-01	1.16E-01	3.74E-01	1.15E-01
EF 3.0 Climate change [kg CO_2_ eq.]	1.47E+09	4.10E+08	4.04E+08	9.68E+08	4.08E+08
EF 3.0 Ecotoxicity freshwater [CTUe]	1.56E+10	7.32E+09	8.05E+09	1.47E+10	1.07E+10
EF 3.0 Eutrophication freshwater [kg P eq.]	4.94E+04	3.43E+04	2.59E+04	4.93E+04	3.72E+04
EF 3.0 Ionising radiation – human health [kBq U235 eq.]	8.05E+07	2.37E+07	9.10E+06	5.34E+07	1.59E+07
EF 3.0 Land use [Pt]	4.68E+09	7.56E+09	3.08E+09	4.45E+09	3.71E+09
EF 3.0 Non-cancer human health effects [CTUh]	7.63E+00	1.55E+01	4.28E+00	6.06E+00	4.44E+00
EF 3.0 Ozone depletion [kg CFC-11 eq.]	2.60E+02	3.92E+01	1.64E+01	1.49E+02	1.97E+01
EF 3.0 Photochemical ozone formation – human health [kg NMVOC eq.]	6.10E+06	1.46E+06	8.37E+05	3.64E+06	8.45E+05
EF 3.0 Resource use, energy carriers [MJ]	2.15E+10	5.37E+09	6.43E+09	1.46E+10	6.68E+09
EF 3.0 Resource use, mineral and metals [kg Sb eq.]	4.87E+02	4.75E+02	4.17E+02	4.77E+02	4.52E+02
EF 3.0 Respiratory inorganics [Disease incidences]	3.23E+01	4.54E+01	1.39E+01	2.06E+01	1.27E+01
EF 3.0 Water scarcity [m^3^ world equiv.]	1.40E+08	1.66E+09	7.02E+07	1.03E+08	6.27E+07

Green indicates the lowest results generated; red indicates the highest results generated.

Hot-spot analyses were carried out on the Climate Change results for Scenario 1a–1c, and were compared with all the other face mask use scenarios. Figure A4 shows that the emissions associated with Mask Transport to the UK are significantly reduced by relocating single-use face mask manufacturing to Turkey. It also illustrates that the impacts generated from Mask Manufacture for Scenarios 1, 1a and 1c are similar, and are lower than in Scenario 1b. Further analysis showed that the difference in Mask Manufacture results (for Scenario 1 and the sub-scenarios) is dependent on the delivery of materials for face mask production. Scenario 1b assumed that the materials would be sourced and imported from China. This showed that the transportation of materials to the UK by airfreight contributes 71.3% towards the Mask Manufacture Climate Change value (57.9% of total impact). The materials transport for face mask manufacture contributed 0.193%, 0.201%, and 8.98% towards the Mask Manufacture Climate Change results for Scenarios 1, 1a and 1c, respectively.

Figure A4 also suggests that if single-use face masks are manufactured in Turkey and the UK (with materials sourced in Turkey), then machine washing reusable face masks (Scenario 4) is the only scenario where reusable masks are environmentally comparable for UK-wide use. The manual washing of face masks without the use of single-use filters (Scenario 2) and the machine washing of face masks with the use of single-use filters (Scenario 5) are only preferable to single-use face masks if the materials are supplied from China.

#### Limitations and discussion

The comparative study explored the environmental impact differences between using a face mask that is designed to be disposed of after one use with different scenarios in which face masks are designed to be washed and reused. The reusing of single-use face masks was not analysed. This is because there are currently no protocols for reusing face masks designed to be used once. Hence, not all face mask use scenarios were explored as part of this study. Equally, a limitation of this comparative study is the washing of face masks. Different techniques may be employed at individual households; for instance, cold washing and other machine-washing techniques. It is acknowledged that cold washing will reduce the thermal energy use to heat water in Scenarios 2 and 3. However, the current guidelines for eliminating viruses suggest the use of hot water and soap. More data is needed about the effectiveness of using cold water and soap for removing viruses before this recommendation can be made.

In Scenarios 2–5, each face mask was assumed to withstand 30–50 washes. It is acknowledged that the products may not withstand this amount of washing, or may be misplaced or damaged by other means. Walser et al. [26] discussed the life of t-shirts and stated that if a garment is washed with low efficacy then its life is 20 washes, 50 washes for medium efficacy and 100 washes for high efficacy. If a face mask has a life of 20 washes, then 18 masks are necessary for one full year of face mask use.

Further analysis was carried out on Scenario 4 to understand the environmental impact of additional supplies of masks. Assuming that the total amount of machine washes per year stays constant and filters continue not to be used, then up to 48 reusable face masks (44 additional masks) can be supplied per person before the impact on Climate Change exceeds the generated value for Scenario 1. Table A14 highlights the maximum number of reusable masks per person for all other environmental impact categories and the average limit is calculated to be 25. Thus, depending on which impact category is of most interest, an additional 21 masks can be supplied over 1 year of mask use, such that Scenario 4 retains its environmental superiority over Scenario 1 (single-mask use). With 25 masks, this reduces the amount of washing per mask to 15 washes, which is below the lower limit of a t-shirt life stated by Walser et al. [26].

**Table A14.** The maximum number of reusable masks in use per person per year (without additional filter use) before the environmental impact exceeds the generated value of using single-use masks (Scenario 1).

Impact category	Number of reusable masks per person
EF 3.0 Acidification terrestrial and freshwater [mole of H+ eq.]	30
EF 3.0 Cancer human health effects [CTUh]	24
EF 3.0 Climate change [kg CO_2_ eq.]	48
EF 3.0 Ecotoxicity freshwater [CTUe]	30
EF 3.0 Eutrophication freshwater [kg P eq.]	20
EF 3.0 Ionising radiation - human health [kBq U235 eq.]	59
EF 3.0 Land use [Pt]	8
EF 3.0 Non-cancer human health effects [CTUh]	5
EF 3.0 Ozone depletion [kg CFC-11 eq.]	82
EF 3.0 Photochemical ozone formation – human health [kg NMVOC eq.]	53
EF 3.0 Resource use, energy carriers [MJ]	54
EF 3.0 Resource use, mineral and metals [kg Sb eq.]	14
EF 3.0 Respiratory inorganics [Disease incidences]	8
EF 3.0 Water scarcity [m^3^ world equiv.]	N/A
**Average**	**25**

Single-use face masks were modelled as being manufactured in China, Turkey and the UK in order to explore the potential reduction in environmental impacts if production was relocated away from China. Reusable face masks (Scenario 2–5) were also assumed to be imported from China, but their production in Turkey or the UK was not modelled. This was because the environmental impact results for reusable Mask Manufacture and Mask Transport to the UK were over 70% lower than those generated for single-use face masks (Scenario 1) for most impact categories. However, there is advice available on how to make reusable face masks at home and therefore the manufacture of face masks in China may not be necessary. This can reduce the overall environmental impact of all reusable face mask scenarios especially if masks are made with waste clothing.

The manufacturing waste that would arise and the associated waste disposal treatments were not modelled in this comparative study, due to the limited data available. However, the percentage contribution of mask waste disposal towards each impact category is low for all scenarios (average percentage contribution <1%) (Appendix 4). From this, it was inferred that the percentage contribution from manufacturing waste treatment should be negligible.

Lastly, this study assumes that every face mask scenario has an equal functionality in preventing the transmission of infection. The effectiveness of face mask use cannot be evaluated using life cycle assessment. A highly developed review by MacIntyre and Chugtai [14] suggests that the effectiveness of face masks in providing protection against infections is subject to compliance, complementary interventions and early use. Thus, although reusable face masks are said to be less effective in a high-risk setting, when used as a precautionary intervention in conjunction with social distancing and regular hand washing, they should have the same effect as single-use face masks.

#### Conclusion

The comparative study results show that using a higher number of reusable face masks, in rotation to allow for machine washing, is the most favourable method of using face masks from an environmental perspective. The use of filters with reusable face masks is discouraged, but can generate a lower environmental impact when compared to single-use face masks if face masks are machine-washed.

Currently, sourcing materials and face masks from China are deemed the most realistic option. However, analyses show that if the manufacture of single-use face masks can be relocated to Turkey and the UK then the environment impact of using of single-use masks in the UK will reduce, but using reusable face masks in rotation and machine washing them (Scenario 4) is still preferable for the environment.

### Appendix 4

**Table A15.** Stage contribution to each environmental impact category for Scenario 1 – using single-use masks in the UK (manufactured in China) (Appendix 4).

	Mask manufacture	Filter manufacture	Mask packaging manufacture	Mask transport to UK	Filter transport to UK	Mask UK distribution	Filter UK distribution	Mask use	Mask waste transport	Filter waste transport	Packaging waste transport	Mask disposal	Filter disposal	Packaging waste disposal
EF 3.0 Acidification terrestrial and freshwater [mole of H+ eq.]	11.35%	0.00%	2.68%	84.31%	0.00%	1.59%	0.00%	0.00%	0.02%	0.00%	0.02%	0.03%	0.00%	0.10%
EF 3.0 Cancer human health effects [CTUh]	12.52%	0.00%	4.39%	80.79%	0.00%	2.44%	0.00%	0.00%	0.01%	0.00%	0.01%	−0.17%	0.00%	−0.01%
EF 3.0 Climate change [kg CO_2_ eq.]	15.56%	0.00%	2.53%	74.38%	0.00%	2.34%	0.00%	0.00%	0.12%	0.00%	0.11%	4.96%	0.00%	1.28%
EF 3.0 Ecotoxicity freshwater [CTUe]	17.81%	0.00%	30.38%	50.16%	0.00%	2.56%	0.00%	0.00%	0.02%	0.00%	0.02%	−0.94%	0.00%	−0.27%
EF 3.0 Eutrophication freshwater [kg P eq.]	10.21%	0.00%	30.25%	51.15%	0.00%	7.63%	0.00%	0.00%	0.00%	0.00%	0.00%	0.76%	0.00%	0.40%
EF 3.0 Ionising radiation - human health [kBq U235 eq.]	11.18%	0.00%	2.14%	88.63%	0.00%	2.97%	0.00%	0.00%	0.00%	0.00%	0.00%	−4.92%	0.00%	−1.83%
EF 3.0 Land use [Pt]	12.29%	0.00%	41.54%	41.96%	0.00%	5.11%	0.00%	0.00%	0.00%	0.00%	0.00%	−0.90%	0.00%	−0.30%
EF 3.0 Non-cancer human health effects [CTUh]	41.32%	0.00%	7.98%	45.32%	0.00%	5.17%	0.00%	0.00%	0.04%	0.00%	0.03%	0.15%	0.00%	1.67%
EF 3.0 Ozone depletion [kg CFC-11 eq.]	0.37%	0.00%	1.21%	95.62%	0.00%	2.80%	0.00%	0.00%	0.00%	0.00%	0.00%	0.00%	0.00%	0.00%
EF 3.0 Photochemical ozone formation – human health [kg NMVOC eq.]	9.45%	0.00%	1.89%	87.41%	0.00%	1.21%	0.00%	0.00%	0.02%	0.00%	0.02%	0.01%	0.00%	0.29%
EF 3.0 Resource use, energy carriers [MJ]	25.51%	0.00%	2.31%	70.92%	0.00%	2.35%	0.00%	0.00%	0.11%	0.00%	0.10%	−1.30%	0.00%	−0.82%
EF 3.0 Resource use, mineral and metals [kg Sb eq.]	26.80%	0.00%	14.07%	22.30%	0.00%	37.64%	0.00%	0.00%	0.00%	0.00%	0.00%	−0.82%	0.00%	−0.32%
EF 3.0 Respiratory inorganics [Disease incidences]	30.34%	0.00%	10.11%	54.60%	0.00%	5.12%	0.00%	0.00%	0.02%	0.00%	0.02%	-0.21%	0.00%	0.01%
EF 3.0 Water scarcity [m^3^ world equiv.]	23.68%	0.00%	8.24%	58.86%	0.00%	3.15%	0.00%	0.00%	0.00%	0.00%	0.00%	6.08%	0.00%	4.16%

**Table A16.** Stage contribution to each environmental impact category for Scenario 2 – using reusable masks, manually washed in the UK.

	Mask manufacture	Filter manufacture	Mask packaging manufacture	Mask transport to UK	Filter transport to UK	Mask UK distribution	Filter UK distribution	Mask use	Mask waste transport	Filter waste Transport	Packaging waste transport	Mask disposal	Filter disposal	Packaging waste disposal
EF 3.0 Acidification terrestrial and freshwater [mole of H+ eq.]	34.34%	0.00%	0.32%	13.17%	0.00%	0.34%	0.00%	51.68%	0.00%	0.00%	0.00%	0.14%	0.00%	0.00%
EF 3.0 Cancer human health effects [CTUh]	25.67%	0.00%	0.30%	7.21%	0.00%	0.30%	0.00%	66.50%	0.00%	0.00%	0.00%	0.02%	0.00%	−0.01%
EF 3.0 Climate change [kg CO_2_ eq.]	11.27%	0.00%	0.53%	7.73%	0.00%	0.34%	0.00%	79.76%	0.02%	0.00%	0.01%	0.35%	0.00%	0.19%
EF 3.0 Ecotoxicity freshwater [CTUe]	23.00%	0.00%	1.06%	4.94%	0.00%	0.35%	0.00%	70.68%	0.00%	0.00%	0.00%	−0.03%	0.00%	−0.03%
EF 3.0 Eutrophication freshwater [kg P eq.]	21.10%	0.00%	0.31%	2.79%	0.00%	0.57%	0.00%	75.17%	0.00%	0.00%	0.00%	0.06%	0.00%	0.02%
EF 3.0 Ionising radiation – human health [kBq U235 eq.]	16.94%	0.00%	0.77%	23.71%	0.00%	1.10%	0.00%	58.60%	0.00%	0.00%	0.00%	−1.11%	0.00%	−0.52%
EF 3.0 Land use [Pt]	33.31%	0.00%	0.27%	1.34%	0.00%	0.23%	0.00%	64.88%	0.00%	0.00%	0.00%	−0.02%	0.00%	−0.01%
EF 3.0 Non-cancer human health effects [CTUh]	54.35%	0.00%	0.30%	1.69%	0.00%	0.27%	0.00%	43.19%	0.00%	0.00%	0.00%	0.20%	0.00%	0.01%
EF 3.0 Ozone depletion [kg CFC-11 eq.]	7.66%	0.00%	0.12%	53.08%	0.00%	2.16%	0.00%	36.98%	0.00%	0.00%	0.00%	0.00%	0.00%	0.00%
EF 3.0 Photochemical ozone formation – human health [kg NMVOC eq.]	20.50%	0.00%	0.50%	21.86%	0.00%	0.42%	0.00%	56.35%	0.01%	0.00%	0.00%	0.36%	0.00%	0.01%
EF 3.0 Resource use, energy carriers [MJ]	9.44%	0.00%	0.99%	7.70%	0.00%	0.35%	0.00%	81.69%	0.02%	0.00%	0.01%	−0.21%	0.00%	−0.06%
EF 3.0 Resource use, mineral and metals [kg Sb eq.]	22.80%	0.00%	0.42%	0.93%	0.00%	2.18%	0.00%	73.70%	0.00%	0.00%	0.00%	−0.03%	0.00%	−0.01%
EF 3.0 Respiratory inorganics [disease incidences]	51.66%	0.00%	0.23%	2.87%	0.00%	0.37%	0.00%	44.87%	0.00%	0.00%	0.00%	0.00%	0.00%	0.00%
EF 3.0 Water scarcity [m^3^ world equiv.]	20.59%	0.00%	0.02%	0.15%	0.00%	0.01%	0.00%	79.20%	0.00%	0.00%	0.00%	0.02%	0.00%	0.01%

**Table A17.** Stage contribution to each environmental impact category for Scenario 3 – using reusable masks with single use filters, manually washed in the UK.

	Mask manufacture	Filter manufacture	Mask packaging manufacture	Mask transport to UK	Filter transport to UK	Mask UK distribution	Filter UK distribution	Mask use	Mask waste transport	Filter waste transport	Packaging waste transport	Mask disposal	Filter disposal	Packaging waste disposal
EF 3.0 Acidification terrestrial and freshwater [mole of H+ eq.]	19.96%	8.38%	1.17%	2.19%	37.07%	0.20%	0.87%	30.04%	0.00%	0.01%	0.01%	0.08%	0.01%	0.03%
EF 3.0 Cancer human health effects [CTUh]	18.12%	6.64%	1.33%	1.45%	24.43%	0.21%	0.92%	46.94%	0.00%	0.00%	0.00%	0.01%	−0.06%	−0.01%
EF 3.0 Climate change [kg CO_2_ eq.]	7.24%	11.14%	1.57%	1.42%	23.97%	0.22%	0.93%	51.22%	0.01%	0.05%	0.03%	0.23%	1.98%	0.61%
EF 3.0 Ecotoxicity freshwater [CTUe]	15.90%	9.99%	6.94%	0.98%	16.43%	0.24%	1.04%	48.88%	0.00%	0.01%	0.01%	−0.02%	−0.38%	−0.12%
EF 3.0 Eutrophication freshwater [kg P eq.]	17.07%	4.08%	3.67%	0.65%	10.90%	0.46%	2.01%	60.91%	0.00%	0.00%	0.00%	0.05%	0.20%	0.09%
EF 3.0 Ionising radiation – human health [kBq U235 eq.]	6.99%	18.71%	1.61%	2.78%	47.11%	0.45%	1.95%	24.08%	0.00%	0.00%	0.00%	−0.46%	−3.24%	−1.17%
EF 3.0 Land use [Pt]	28.56%	6.03%	3.18%	0.33%	5.52%	0.19%	0.84%	55.52%	0.00%	0.00%	0.00%	−0.02%	−0.15%	−0.05%
EF 3.0 Non-cancer human health effects [CTUh]	44.36%	10.55%	1.38%	0.39%	6.66%	0.22%	0.94%	35.30%	0.00%	0.01%	0.00%	0.16%	0.03%	0.14%
EF 3.0 Ozone depletion [kg CFC-11 eq.]	2.32%	0.49%	0.56%	4.59%	77.40%	0.65%	2.81%	11.19%	0.00%	0.00%	0.00%	0.00%	0.00%	0.00%
EF 3.0 Photochemical ozone formation – human health [kg NMVOC eq.]	9.55%	9.85%	1.30%	2.90%	48.98%	0.20%	0.84%	26.19%	0.00%	0.01%	0.01%	0.17%	0.00%	0.09%
EF 3.0 Resource use, energy carriers [MJ]	5.97%	14.37%	2.57%	1.39%	23.45%	0.22%	0.97%	51.64%	0.01%	0.05%	0.03%	−0.13%	−0.54%	−0.26%
EF 3.0 Resource use, mineral and metals [kg Sb eq.]	17.85%	9.69%	2.14%	0.21%	3.52%	1.71%	7.37%	57.70%	0.00%	0.00%	0.00%	−0.02%	−0.16%	−0.06%
EF 3.0 Respiratory inorganics [disease incidences]	41.15%	8.38%	1.53%	0.65%	11.01%	0.30%	1.28%	35.73%	0.00%	0.01%	0.00%	0.00%	−0.05%	−0.01%
EF 3.0 Water scarcity [m^3^ world equiv.]	20.37%	0.31%	0.13%	0.04%	0.73%	0.01%	0.05%	78.24%	0.00%	0.00%	0.00%	0.02%	0.09%	0.05%

**Table A18.** Stage contribution to each environmental impact category for Scenario 4 – using reusable masks, machine washed in the UK.

	Mask manufacture	Filter manufacture	Mask packaging manufacture	Mask transport to UK	Filter transport to UK	Mask UK distribution	Filter UK distribution	Mask use	Mask waste transport	Filter waste transport	Packaging waste transport	Mask disposal	Filter disposal	Packaging waste disposal
EF 3.0 Acidification terrestrial and freshwater [mole of H+ eq.]	66.10%	0.00%	0.62%	25.45%	0.00%	0.66%	0.00%	6.89%	0.01%	0.00%	0.00%	0.26%	0.00%	0.01%
EF 3.0 Cancer human health effects [CTUh]	9.61%	0.00%	0.11%	2.69%	42.83%	0.11%	42.83%	1.79%	0.00%	0.00%	0.00%	0.01%	0.00%	0.00%
EF 3.0 Climate change [kg CO_2_ eq.]	48.85%	0.00%	2.31%	33.51%	0.00%	1.46%	0.00%	12.25%	0.08%	0.00%	0.03%	1.51%	0.00%	0.83%
EF 3.0 Ecotoxicity freshwater [CTUe]	63.81%	0.00%	2.95%	13.69%	0.00%	0.97%	0.00%	18.66%	0.01%	0.00%	0.00%	−0.09%	0.00%	−0.10%
EF 3.0 Eutrophication freshwater [kg P eq.]	70.62%	0.00%	1.04%	9.35%	0.04%	1.93%	0.04%	16.79%	0.00%	0.00%	0.00%	0.20%	0.00%	0.06%
EF 3.0 Ionising radiation – human health [kBq U235 eq.]	27.97%	0.00%	1.26%	39.09%	0.00%	1.81%	0.00%	31.70%	0.00%	0.00%	0.00%	−1.83%	0.00%	−0.85%
EF 3.0 Land use [Pt]	82.70%	0.00%	0.66%	3.32%	0.00%	0.56%	0.00%	12.82%	0.00%	0.00%	0.00%	−0.06%	0.00%	−0.03%
EF 3.0 Non-cancer human health effects [CTUh]	26.98%	0.00%	0.15%	0.84%	34.92%	0.13%	34.92%	1.95%	0.00%	0.00%	0.00%	0.10%	0.00%	0.01%
EF 3.0 Ozone depletion [kg CFC-11 eq.]	5.50%	0.00%	0.09%	38.13%	26.44%	1.55%	26.44%	1.86%	0.00%	0.00%	0.00%	0.00%	0.00%	0.00%
EF 3.0 Photochemical ozone formation – human health [kg NMVOC eq.]	43.63%	0.00%	1.07%	46.41%	0.00%	0.89%	0.00%	7.22%	0.01%	0.00%	0.00%	0.76%	0.00%	0.03%
EF 3.0 Resource use, energy carriers [MJ]	44.82%	0.00%	4.71%	36.53%	0.00%	1.68%	0.00%	13.14%	0.08%	0.00%	0.03%	−0.98%	0.00%	−0.29%
EF 3.0 Resource use, mineral and metals [kg Sb eq.]	64.57%	0.00%	1.20%	2.64%	4.76%	6.17%	4.76%	15.99%	0.00%	0.00%	0.00%	−0.09%	0.00%	−0.04%
EF 3.0 Respiratory inorganics [Disease incidences]	41.52%	0.00%	0.18%	2.30%	26.69%	0.30%	26.69%	2.31%	0.00%	0.00%	0.00%	0.00%	0.00%	0.00%
EF 3.0 Water scarcity [m^3^ world equiv.]	81.33%	0.00%	0.09%	0.61%	0.00%	0.04%	0.00%	17.85%	0.00%	0.00%	0.00%	0.08%	0.00%	0.03%

**Table A19.** Stage contribution to each environmental impact category for Scenario 5 – using reusable masks with single use filters, machine-washed in the UK.

	Mask manufacture	Filter manufacture	Mask packaging manufacture	Mask transport to UK	Filter transport to UK	Mask UK distribution	Filter UK distribution	Mask use	Mask waste transport	Filter waste transport	Packaging waste transport	Mask disposal	Filter disposal	Packaging waste disposal
EF 3.0 Acidification terrestrial and freshwater [mole of H+ Eq.]	37.95%	9.32%	1.25%	14.61%	31.35%	0.38%	0.96%	3.96%	0.01%	0.01%	0.01%	0.15%	0.02%	0.03%
EF 3.0 Cancer human health effects [CTUh]	42.34%	9.05%	1.74%	11.85%	25.43%	0.50%	1.25%	7.90%	0.00%	0.01%	0.00%	0.03%	−0.09%	−0.02%
EF 3.0 Climate change [kg CO_2_ eq.]	20.87%	18.78%	2.59%	14.32%	30.72%	0.62%	1.57%	5.24%	0.03%	0.08%	0.05%	0.65%	3.33%	1.16%
EF 3.0 Ecotoxicity freshwater [CTUe]	38.39%	14.03%	9.16%	8.24%	17.67%	0.58%	1.46%	11.22%	0.00%	0.01%	0.01%	−0.06%	−0.54%	−0.20%
EF 3.0 Eutrophication freshwater [kg P eq.]	49.54%	6.90%	5.75%	6.56%	14.07%	1.35%	3.41%	11.78%	0.00%	0.00%	0.00%	0.14%	0.34%	0.16%
EF 3.0 Ionising radiation – human health [kBq U235 eq.]	12.49%	19.51%	1.63%	17.46%	37.46%	0.81%	2.04%	14.16%	0.00%	0.00%	0.00%	−0.82%	−3.37%	−1.36%
EF 3.0 Land use [Pt]	67.40%	8.30%	4.06%	2.70%	5.80%	0.46%	1.15%	10.45%	0.00%	0.00%	0.00%	−0.05%	−0.20%	−0.08%
EF 3.0 Non-cancer human health effects [CTUh]	74.07%	10.28%	1.29%	2.31%	4.95%	0.36%	0.92%	5.36%	0.00%	0.01%	0.00%	0.27%	0.03%	0.15%
EF 3.0 Ozone depletion [kg CFC-11 eq.]	4.10%	0.51%	0.53%	28.43%	60.99%	1.15%	2.90%	1.39%	0.00%	0.00%	0.00%	0.00%	0.00%	0.00%
EF 3.0 Photochemical ozone formation – human health [kg NMVOC eq.]	18.91%	11.39%	1.44%	20.12%	43.17%	0.39%	0.97%	3.13%	0.01%	0.02%	0.01%	0.33%	0.00%	0.11%
EF 3.0 Resource use, energy carriers [MJ]	18.03%	25.30%	4.46%	14.69%	31.52%	0.67%	1.70%	5.29%	0.03%	0.08%	0.05%	−0.39%	−0.94%	−0.50%
EF 3.0 Resource use, mineral and metals [kg Sb eq.]	47.91%	15.17%	3.20%	1.96%	4.21%	4.58%	11.53%	11.86%	0.00%	0.00%	0.00%	−0.06%	−0.25%	−0.10%
EF 3.0 Respiratory inorganics [disease incidences]	71.73%	8.52%	1.47%	3.98%	8.54%	0.52%	1.30%	3.99%	0.00%	0.01%	0.00%	0.01%	−0.05%	−0.01%
EF 3.0 Water scarcity [m^3^ world equiv.]	79.21%	0.71%	0.28%	0.59%	1.27%	0.04%	0.11%	17.39%	0.00%	0.00%	0.00%	0.07%	0.21%	0.12%

**Table A20.** Stage contribution to each environmental impact category for Scenario 1a – using single-use masks in the UK (mask manufactured in Turkey).

	Mask manufacture	Filter manufacture	Mask packaging manufacture	Mask transport to UK	Filter transport to UK	Mask UK distribution	Filter UK distribution	Mask use	Mask waste transport	Filter waste transport	Packaging waste transport	Mask disposal	Filter disposal	Packaging waste disposal
EF 3.0 Acidification terrestrial and freshwater [mole of H+ eq.]	70.96%	0.00%	14.19%	5.56%	0.00%	8.41%	0.00%	0.00%	0.12%	0.00%	0.10%	0.14%	0.00%	0.53%
EF 3.0 Cancer human health effects [CTUh]	59.80%	0.00%	23.04%	5.16%	0.00%	12.86%	0.00%	0.00%	0.06%	0.00%	0.05%	−0.89%	0.00%	−0.07%
EF 3.0 Climate change [kg CO_2_ eq.]	54.22%	0.00%	9.09%	5.03%	0.00%	8.42%	0.00%	0.00%	0.44%	0.00%	0.38%	17.82%	0.00%	4.60%
EF 3.0 Ecotoxicity freshwater [CTUe]	35.28%	0.00%	59.01%	3.02%	0.00%	4.98%	0.00%	0.00%	0.04%	0.00%	0.04%	−1.84%	0.00%	−0.53%
EF 3.0 Eutrophication freshwater [kg P eq.]	19.46%	0.00%	57.48%	6.34%	0.00%	14.53%	0.00%	0.00%	0.00%	0.00%	0.00%	1.44%	0.00%	0.76%
EF 3.0 Ionising radiation – human health [kBq U235 eq.]	97.11%	0.00%	19.25%	17.71%	0.00%	26.73%	0.00%	0.00%	0.03%	0.00%	0.03%	−44.33%	0.00%	−16.53%
EF 3.0 Land use [Pt]	18.38%	0.00%	63.61%	12.05%	0.00%	7.79%	0.00%	0.00%	0.00%	0.00%	0.00%	−1.38%	0.00%	−0.45%
EF 3.0 Non-cancer human health effects [CTUh]	67.96%	0.00%	14.08%	5.60%	0.00%	9.06%	0.00%	0.00%	0.06%	0.00%	0.05%	0.26%	0.00%	2.92%
EF 3.0 Ozone depletion [kg CFC-11 eq.]	5.84%	0.00%	19.10%	30.66%	0.00%	44.41%	0.00%	0.00%	0.00%	0.00%	0.00%	0.00%	0.00%	0.00%
EF 3.0 Photochemical ozone formation – human health [kg NMVOC eq.]	67.84%	0.00%	13.78%	7.19%	0.00%	8.81%	0.00%	0.00%	0.14%	0.00%	0.12%	0.04%	0.00%	2.08%
EF 3.0 Resource use, energy carriers [MJ]	85.48%	0.00%	7.78%	5.24%	0.00%	7.94%	0.00%	0.00%	0.38%	0.00%	0.33%	−4.41%	0.00%	−2.75%
EF 3.0 Resource use, mineral and metals [kg Sb eq.]	31.33%	0.00%	16.52%	9.35%	0.00%	44.13%	0.00%	0.00%	0.01%	0.00%	0.00%	−0.96%	0.00%	−0.38%
EF 3.0 Respiratory inorganics [disease incidences]	52.41%	0.00%	23.40%	12.70%	0.00%	11.84%	0.00%	0.00%	0.05%	0.00%	0.05%	−0.48%	0.00%	0.03%
EF 3.0 Water scarcity [m^3^ world equiv.]	54.49%	0.00%	15.79%	4.02%	0.00%	6.04%	0.00%	0.00%	0.00%	0.00%	0.00%	11.67%	0.00%	7.98%

**Table A21.** Stage contribution to each environmental impact category for Scenario 1b – Using single-use masks in the UK (mask manufactured in UK, materials imported from China).

	Mask manufacture	Filter manufacture	Mask packaging manufacture	Mask transport to UK	Filter transport to UK	Mask UK distribution	Filter UK distribution	Mask use	Mask waste transport	Filter waste transport	Packaging waste transport	Mask disposal	Filter disposal	Packaging waste disposal
EF 3.0 Acidification terrestrial and freshwater [mole of H+ eq.]	90.21%	0.00%	6.89%	0.00%	0.00%	2.62%	0.00%	0.00%	0.04%	0.00%	0.03%	0.04%	0.00%	0.16%
EF 3.0 Cancer human health effects [CTUh]	88.79%	0.00%	7.49%	0.00%	0.00%	3.98%	0.00%	0.00%	0.02%	0.00%	0.02%	−0.28%	0.00%	−0.02%
EF 3.0 Climate change [kg CO_2_ eq.]	81.26%	0.00%	5.53%	0.00%	0.00%	3.51%	0.00%	0.00%	0.18%	0.00%	0.16%	7.43%	0.00%	1.92%
EF 3.0 Ecotoxicity freshwater [CTUe]	48.41%	0.00%	50.12%	0.00%	0.00%	2.73%	0.00%	0.00%	0.02%	0.00%	0.02%	−1.01%	0.00%	−0.29%
EF 3.0 Eutrophication freshwater [kg P eq.]	38.11%	0.00%	53.11%	0.00%	0.00%	7.62%	0.00%	0.00%	0.00%	0.00%	0.00%	0.75%	0.00%	0.40%
EF 3.0 Ionising radiation – human health [kBq U235 eq.]	93.98%	0.00%	11.81%	0.00%	0.00%	4.55%	0.00%	0.00%	0.01%	0.00%	0.00%	−7.54%	0.00%	−2.81%
EF 3.0 Land use [Pt]	37.95%	0.00%	57.93%	0.00%	0.00%	5.39%	0.00%	0.00%	0.00%	0.00%	0.00%	−0.95%	0.00%	−0.31%
EF 3.0 Non-cancer human health effects [CTUh]	77.80%	0.00%	13.46%	0.00%	0.00%	6.41%	0.00%	0.00%	0.04%	0.00%	0.04%	0.18%	0.00%	2.06%
EF 3.0 Ozone depletion [kg CFC-11 eq.]	90.81%	0.00%	4.28%	0.00%	0.00%	4.91%	0.00%	0.00%	0.00%	0.00%	0.00%	0.00%	0.00%	0.00%
EF 3.0 Photochemical ozone formation – human health [kg NMVOC eq.]	92.59%	0.00%	4.84%	0.00%	0.00%	2.02%	0.00%	0.00%	0.03%	0.00%	0.03%	0.01%	0.00%	0.48%
EF 3.0 Resource use, energy carriers [MJ]	93.75%	0.00%	5.59%	0.00%	0.00%	3.50%	0.00%	0.00%	0.17%	0.00%	0.15%	−1.94%	0.00%	−1.21%
EF 3.0 Resource use, mineral and metals [kg Sb eq.]	41.05%	0.00%	21.57%	0.00%	0.00%	38.54%	0.00%	0.00%	0.00%	0.00%	0.00%	−0.84%	0.00%	−0.33%
EF 3.0 Respiratory inorganics [disease incidences]	71.53%	0.00%	20.68%	0.00%	0.00%	8.03%	0.00%	0.00%	0.04%	0.00%	0.03%	−0.33%	0.00%	0.02%
EF 3.0 Water scarcity [m^3^ world equiv.]	63.76%	0.00%	18.69%	0.00%	0.00%	4.13%	0.00%	0.00%	0.00%	0.00%	0.00%	7.97%	0.00%	5.45%

**Table A22.** Stage contribution to each environmental impact category for Scenario 1c – using single-use masks in the UK (mask manufactured in UK, materials imported from Turkey).

	Mask manufacture	Filter manufacture	Mask packaging manufacture	Mask transport to UK	Filter transport to UK	Mask UK distribution	Filter UK distribution	Mask use	Mask waste transport	Filter waste transport	Packaging waste transport	Mask disposal	Filter disposal	Packaging waste disposal
EF 3.0 Acidification terrestrial and freshwater [mole of H+ eq.]	63.67%	0.00%	25.59%	0.00%	0.00%	9.73%	0.00%	0.00%	0.13%	0.00%	0.12%	0.16%	0.00%	0.61%
EF 3.0 Cancer human health effects [CTUh]	63.65%	0.00%	24.28%	0.00%	0.00%	12.92%	0.00%	0.00%	0.06%	0.00%	0.05%	−0.89%	0.00%	−0.07%
EF 3.0 Climate change [kg CO_2_ eq.]	55.46%	0.00%	13.15%	0.00%	0.00%	8.34%	0.00%	0.00%	0.44%	0.00%	0.38%	17.67%	0.00%	4.56%
EF 3.0 Ecotoxicity freshwater [CTUe]	29.06%	0.00%	68.91%	0.00%	0.00%	3.76%	0.00%	0.00%	0.03%	0.00%	0.03%	−1.39%	0.00%	−0.40%
EF 3.0 Eutrophication freshwater [kg P eq.]	17.93%	0.00%	70.43%	0.00%	0.00%	10.11%	0.00%	0.00%	0.00%	0.00%	0.00%	1.00%	0.00%	0.53%
EF 3.0 Ionising radiation – human health [kBq U235 eq.]	79.67%	0.00%	39.90%	0.00%	0.00%	15.37%	0.00%	0.00%	0.02%	0.00%	0.02%	−25.48%	0.00%	−9.48%
EF 3.0 Land use [Pt]	25.62%	0.00%	69.43%	0.00%	0.00%	6.46%	0.00%	0.00%	0.00%	0.00%	0.00%	−1.14%	0.00%	−0.38%
EF 3.0 Non-cancer human health effects [CTUh]	69.76%	0.00%	18.34%	0.00%	0.00%	8.73%	0.00%	0.00%	0.06%	0.00%	0.05%	0.25%	0.00%	2.81%
EF 3.0 Ozone depletion [kg CFC-11 eq.]	30.52%	0.00%	32.35%	0.00%	0.00%	37.13%	0.00%	0.00%	0.00%	0.00%	0.00%	0.00%	0.00%	0.00%
EF 3.0 Photochemical ozone formation – human health [kg NMVOC eq.]	68.12%	0.00%	20.81%	0.00%	0.00%	8.72%	0.00%	0.00%	0.14%	0.00%	0.12%	0.04%	0.00%	2.06%
EF 3.0 Resource use, energy carriers [MJ]	86.33%	0.00%	12.22%	0.00%	0.00%	7.65%	0.00%	0.00%	0.37%	0.00%	0.32%	−4.23%	0.00%	−2.65%
EF 3.0 Resource use, mineral and metals [kg Sb eq.]	37.80%	0.00%	22.77%	0.00%	0.00%	40.67%	0.00%	0.00%	0.00%	0.00%	0.00%	−0.89%	0.00%	−0.35%
EF 3.0 Respiratory inorganics [disease incidences]	53.75%	0.00%	33.59%	0.00%	0.00%	13.04%	0.00%	0.00%	0.06%	0.00%	0.05%	−0.53%	0.00%	0.03%
EF 3.0 Water scarcity [m^3^ world equiv.]	40.55%	0.00%	30.65%	0.00%	0.00%	6.77%	0.00%	0.00%	0.00%	0.00%	0.00%	13.08%	0.00%	8.94%
